# Experimental Evolution with *Caenorhabditis* Nematodes

**DOI:** 10.1534/genetics.115.186288

**Published:** 2017

**Authors:** Henrique Teotónio, Suzanne Estes, Patrick C. Phillips, Charles F. Baer

**Affiliations:** * Institut de Biologie de l´École Normale Supérieure (IBENS), Institut National de la Santé et de la Recherche Médicale U1024, Centre Nationnal de la Recherche Scientifique Unité Mixte de Recherche 8197, Paris Sciences et Lettres Research University, 75005 Paris, France; † Department of Biology, Portland State University, Oregon 97201; ‡ Institute of Ecology and Evolution, 5289 University of Oregon, Eugene, Oregon 97403, and; § Department of Biology, and; ** University of Florida Genetics Institute, University of Florida, Gainesville, Florida 32611

**Keywords:** adaptation, *C. briggsae*, *C. elegans*, *C. remanei*, domestication, experimental design, laboratory selection experiments, self-fertilization, reproduction systems, mutation accumulation, standing genetic variation, WormBook

## Abstract

The hermaphroditic nematode *Caenorhabditis elegans* has been one of the primary model systems in biology since the 1970s, but only within the last two decades has this nematode also become a useful model for experimental evolution. Here, we outline the goals and major foci of experimental evolution with *C. elegans* and related species, such as *C. briggsae* and *C. remanei*, by discussing the principles of experimental design, and highlighting the strengths and limitations of *Caenorhabditis* as model systems. We then review three exemplars of *Caenorhabditis* experimental evolution studies, underlining representative evolution experiments that have addressed the: (1) maintenance of genetic variation; (2) role of natural selection during transitions from outcrossing to selfing, as well as the maintenance of mixed breeding modes during evolution; and (3) evolution of phenotypic plasticity and its role in adaptation to variable environments, including host–pathogen coevolution. We conclude by suggesting some future directions for which experimental evolution with *Caenorhabditis* would be particularly informative.

“With them, many important questions will be accessible to patient observers who do not fear long-term experiments.” – Emile Maupas (1900)

OVER a century ago, Emile Maupas introduced the nematode *Caenorhabditis elegans* to the scientific community with his report on a failed experiment aimed at testing the hypothesis that continual self-fertilization (selfing) should lead to population extinction (maupas 1900). This goal was ultimately thwarted, as after nearly 50 generations of selfing, Maupas’ *C. elegans* culture collapsed due to an errant spike in temperature that led to abnormalities in development and reproduction independently of inbreeding effects. Maupas’ experimental evolution study was inspired by an ongoing debate about the long-term sustainability of selfing as a reproductive strategy (darwin 1876), and provides a particularly telling introduction to experimental evolution: the expected outcome (extinction) was achieved, but for the “wrong” reason, as it was not a result of selfing.

Experimental Evolution (EE) has long been used as the gold standard for testing evolutionary hypotheses about natural selection and genetic drift, estimating theoretical parameters regarding standing genetic variation, such as mutation and recombination rates, and, more recently, as a means for gene discovery. The main organismal models to which EE has been applied are mice, fruit flies, yeast, and bacteria (rose and Lauder 1996; bell 1997; garland and Rose 2009; kassen 2014). Despite the promising start by Emile Maupas (Maupas 1900), however, it was nearly 90 years before *Caenorhabditis* reappeared in EE research, during which time much evolutionary theory had been mathematically formalized.

Because of its relative newcomer status in EE research, we have barely begun to tap the potential of *Caenorhabditis* for elucidating the patterns and processes of evolution (gray and Cutter 2014). But, as the community of *Caenorhabditis* evolutionary biologists has grown—now sufficiently large to merit regular meetings and dedicated stock and databases (Supplemental Material, Table S1 in File S1; carvalho *et al.* 2006; haag *et al.* 2007; braendle and Teotónio 2015)—so too has the array of evolutionary problems being investigated with experiments (Table 1 lists some of the studies that will be covered here).

**Table 1:** Selected studies with *Caenorhabditis* EE

Topic	Question	Approach	Key findings	Exemplars
Evolution of reproductive modes	Is androdioecy maintained in unperturbed or mutagenic environments?	Natural selection, imposed by artificially increasing the frequency of males; populations with N2 and other wild isolate backgrounds; track male frequency	Males are selected against	Stewart and Phillips (2002); Manoel *et al.* (2007); Cutter (2005); Chasnov and Chow (2002)
	Is genetic variation for outcrossing performance sufficient to maintain males?	Evolution from standing genetic variation	Partial selfing is maintained	Teotónio *et al.* (2012); Anderson *et al.* (2010)
	What is the role of selection in breeding mode transitions?	Evolution from standing genetic variation or selection on N2 background variants	Reproductive assurance can promote transition to selfing; increased effective recombination promotes transitions to outcrossing	Theologidis *et al.* (2014); Slowinski *et al.* (2016); Wegewitz *et al.* (2009)
	Does coevolution with a pathogen facilitate maintenance of outcrossing?	Evolution from standing genetic variation	Coevolution with a pathogen favors outcrossing	Morran *et al.* (2009b); Morran *et al.* (2011)
Evolution in variable environments	Does host-pathogen coevolution lead to a “geographic mosaic” of local adaptation?	Standing variation; Natural selection imposed by allowing host and pathogen to coevolve; controls allowed to evolve in isolation	Local adaptation varies among replicates in a manner consistent with “Geographic Mosaic” hypothesis Thompson (2005)	Schulte *et al.* (2011); Masri *et al.* (2013)
	Does phenotypic plasticity evolve under inducible heat-shock environments?	Natural selection imposed by applying a sub-lethal heat shock to L1 larvae; Evolution from standing genetic variation	Genetic assimilation evolves rapidly via changes in thermal reaction norms without large changes in transcriptional regulation	Sikkink *et al.* (2014a,b)
	What are the roles of maternal effects in adaptation to fluctuating environments?	Natural selection under regular and irregular normoxia-anoxia environments; Evolution from standing genetic variation but no recombination	Maternal glycogen provisioning increases geometric mean fitness in regular environments; maternal “bet-hedging” does not evolve in irregular environments	Dey *et al.* (2016)
Evolution of life-history	How does sperm morphology evolve in response to sexual selection for increased sperm competition?	Natural selection imposed by enforced outcrossing with a mutation that renders hermaphrodites male-sterile	Sperm size (and thus sperm fitness) increases rapidly in response to selection for sperm competition	LaMunyon and Ward (2002); see also Murray and Cutter (2011)
	Does antagonistic pleiotropy lead to decrease longevity when early fecundity is selected for?	Selection for early reproduction via discrete population transfers	Longevity does not decrease, and in fact can increase, probably due to laboratory adaptation and resolution of nonequilibrium genetic variation in crossed base population	Anderson *et al.* (2011); Carvalho *et al.* (2014)
	Does changing the sex ratio within hermaphrodites increase the likelihood of sexual conflict?	Experimental evolution under increased presence of males (*fog-2*)	Increased sexual conflict as evidenced by increases in mating-induced female mortality	Palopoli *et al.* (2015); Carvalho *et al.* (2014)
	Does mutation provide sufficient genetic variation to permit a response to selection on body size?	Artificial selection for increased and decreased body volume at maturity	Mutational bias for small body size can explain the observed response to selection.	Azevedo *et al.* (2002); Ostrow *et al.* (2007)
Evolution of development	What is the pattern of selection on intracellular traits?	Mutation accumulation and wild isolates; measurement of cell divisions in embryos	Stabilizing selection on embryo size and mutational correlations therewith can explain the observed pattern of standing genetic variation.	Farhadifar *et al.* (2015)
	How strong is selection on canalization of developmental pathways?	Use temperature sensitive mutants (*tra-2*) to perturb sex ratio and observe evolutionary response in sex determination	Poorly performing sexual phenotypes evolve toward wildtype function under compensatory changes	Chandler *et al.* (2012)
	How does hermaphrodite gametogenesis respond to selection for selfing?	100 generations of evolution under partial selfing from standing genetic variation	Increased early fecundity under selfing results in part from a delay in the switch from spermatogenesis and oogenesis	Poullet *et al.* (2016)
	Is there heritability for developmental robustness?	Mutation accumulation; measurement of vulval development in several laboratory environments	The rate and type of mutational variation for vulva development is genotype and species-specific.	Braendle *et al.* (2010)
Population genetics	What are the cumulative effects on fitness of spontaneous mutations?	Mutation accumulation	The rate of mutational decay of fitness in *C. elegans* is ∼10× slower than previous estimates from *Drosophila*	Keightley and Caballero (1997); also see Vassilieva and Lynch (1999) and Baer *et al.* (2005), among others
	How fast can mutationally-degraded populations recover fitness when efficient selection is restored?	Natural selection applied by allowing low-fitness MA lines to evolve at large population size; controls are the unevolved ancestor of the MA lines and the low-fitness MA lines	Fitness recovers consistently and very rapidly (apparently via compensatory evolution), but never exceeds ancestral fitness	Estes and Lynch (2003); Denver *et al.* (2010); Estes *et al.* (2011)
	Can genetic drift be described as a branching process in the absence of selection?	Invasions of mutants into “resident” populations; follow proportion of extinctions	Most beneficial or deleterious mutants will be lost by genetic drift; frequency-dependence may play a role at intermediate allele frequencies	Chelo *et al.* (2013b)
	How does selection operate at multiple levels of biological organization?	MA experiments at variable population sizes; track mitochondrial deletion frequency	Incidence of mitochondrial deletion increases with reduced efficiency of selection at the individual level	Phillips *et al.* (2015)
	What are the relative roles of environmental specialization and sexual selection in generating reproductive isolation	Long term selection of *C. remanei* across different levels of genetic drift and variable environments	Drift and environmental variation did not enhance reproductive isolation, but apparently evolution of sexual interactions within replicates did	Castillo *et al.* (2015)
Domestication	Has long-term laboratory maintenance inadvertently selected for novel traits in the N2 *C. elegans* strain?	Inadvertent natural selection imposed by the community of *C. elegans* researchers	Several genes—*npr-1*, *glb-5*, *nath-10*—evolved novel alleles that encode adaptive phenotypes under laboratory conditions	Summarized in Sterken *et al.* (2015); see also Weber *et al.* (2010)
	What are the consequences of laboratory adaptation from standing genetic variation?	Hybridization of wild isolates followed by 100 generations of evolution at high population sizes and partial selfing	Extensive outbreeding depression is in part resolved by the maintenance of inbreeding depression	Teotónio *et al.* (2012); Chelo *et al.* (2013a); Chelo and Teotónio (2013)

*Caenorhabditis* are free-living bactivorous roundworms with over 25 species currently being cultured in the laboratory (kiontke *et al.* 2011; felix *et al.* 2014), although only *C. elegans*, *C. briggsae*, and *C. remanei* have been utilized in EE research. A distinctive feature of this group of nematodes is that facultative selfing evolved independently from ancestral obligatory outcrossing three times (kiontke and fitch 2005). *C. elegans*, *C. briggsae*, and *C. tropicalis* have a rare androdioecious reproduction system, with hermaphrodites capable of selfing, and of outcrossing with males, but not with other hermaphrodites. Hermaphrodites from these species are developmentally similar to females of related dioecious species, except for a period during germline specification and differentiation when sperm is produced and stored in the spermatheca prior to an irreversible switch to oogenesis at adulthood. These hermaphrodites are therefore self-sperm limited and can only fertilize all of their oocytes when mated by males (barker 1992; cutter 2004). Behaviorally, hermaphrodites have lost the ancestral ability to attract males, and are generally reluctant to mate until they have depleted their own self-sperm store (lipton *et al.* 2004; chasnov *et al.* 2007).

Our aim with this review is to present *Caenorhabditis* species as excellent models for EE. We first focus on the basic principles of EE, which apply more or less to any organism, and then introduce *Caenorhabditis* and related resources for their use in EE. We next explore the common goals and outcomes of EE studies in sections devoted to laboratory domestication and specific EE designs that address the fundamental processes of natural selection, genetic drift, mutation, segregation, and recombination. We then review selected studies that have greatly improved our understanding of several evolutionary problems. More technical introductions are presented in Boxes and Figures, and in Supplementary Appendices. We finish with future research directions for which we believe *Caenorhabditis* to be particularly well-suited as model systems.

## What Is Experimental Evolution?

### Advantages and limitations of the experimental manipulation of evolution

**Figure 1:**
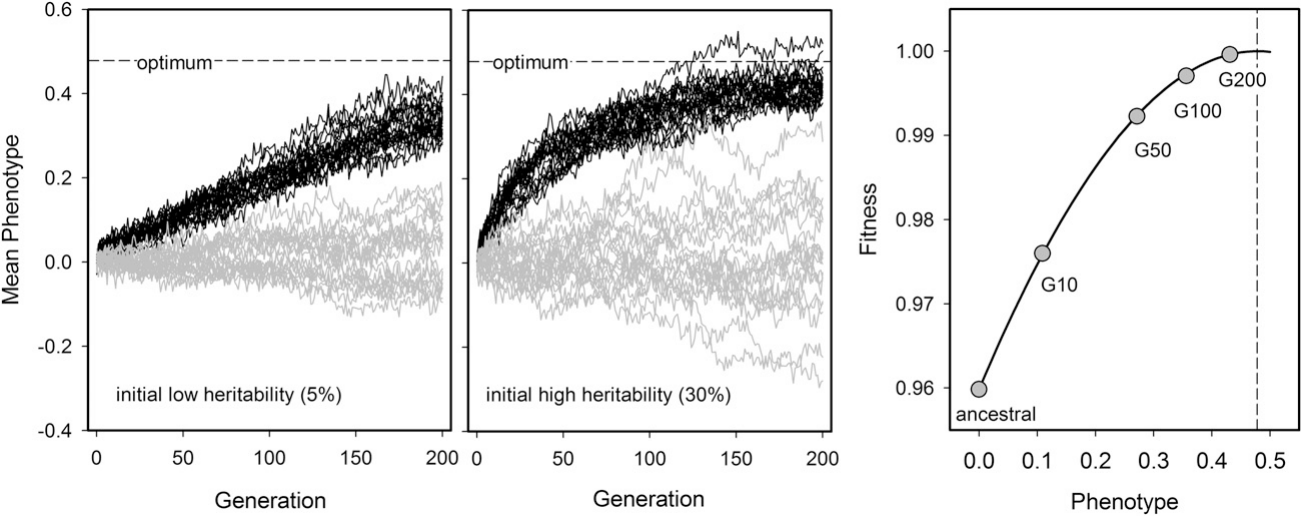
Phenotypic divergence and differentiation in EE. A typical evolution experiment involves manipulation of environmental conditions and letting populations *diverge* from the ancestral population in trait value. To show that divergence is genetically based, one needs to account for confounding environmental effects (including transgenerational carryover effects) by assaying ancestral and derived populations after some number of generations of common “garden” culture. “Control” replicate populations are usually kept in the environment to which the ancestral population has adapted to, that is, the domestication environment, and run alongside those in the new environment such that the mean *differentiation* between treatments is interpreted to result from selection. It is usually assumed that control populations are under no selection, but it is perhaps more realistic to think that they are under stabilizing selection for intermediate trait values. In mutation accumulation (MA) experiments, since selection efficiency is low, a domestication stage prior to subjecting populations to new environmental conditions is not necessary. Any differentiation among replicates within each treatment is presumed to result from genetic drift and idiosyncratic selection (*e.g.*, placement within incubators, the experimenter doing the culturing, etc.). We illustrate divergence and differentiation of a quantitative trait with individual-based simulations (http://datadryad.org/, doi: 10.5061/dryad.bg08n). The plots in the figure show these simulations of divergence and differentiation for 20 populations reproducing exclusively by selfing evolving under genetic drift and no selection (gray lines), or genetic drift and selection (black lines). With lower initial heritability it takes longer for selection to take populations to the new phenotypic optimum (scenario 1; left), and the higher the initial heritability the faster the initial response (scenario 2; middle). In natural populations, lower heritability is typical of behavioral traits, while high heritability is typical of morphological traits (Lynch and Walsh 1998). The phenotypic “optimum” is shown by a dashed line. The right plot shows the fitness function employed for the simulations, with the circles illustrating the mean evolution of the selected populations starting with high heritability. Further details about the simulation can be found in Figure 2.

**Figure 2:**
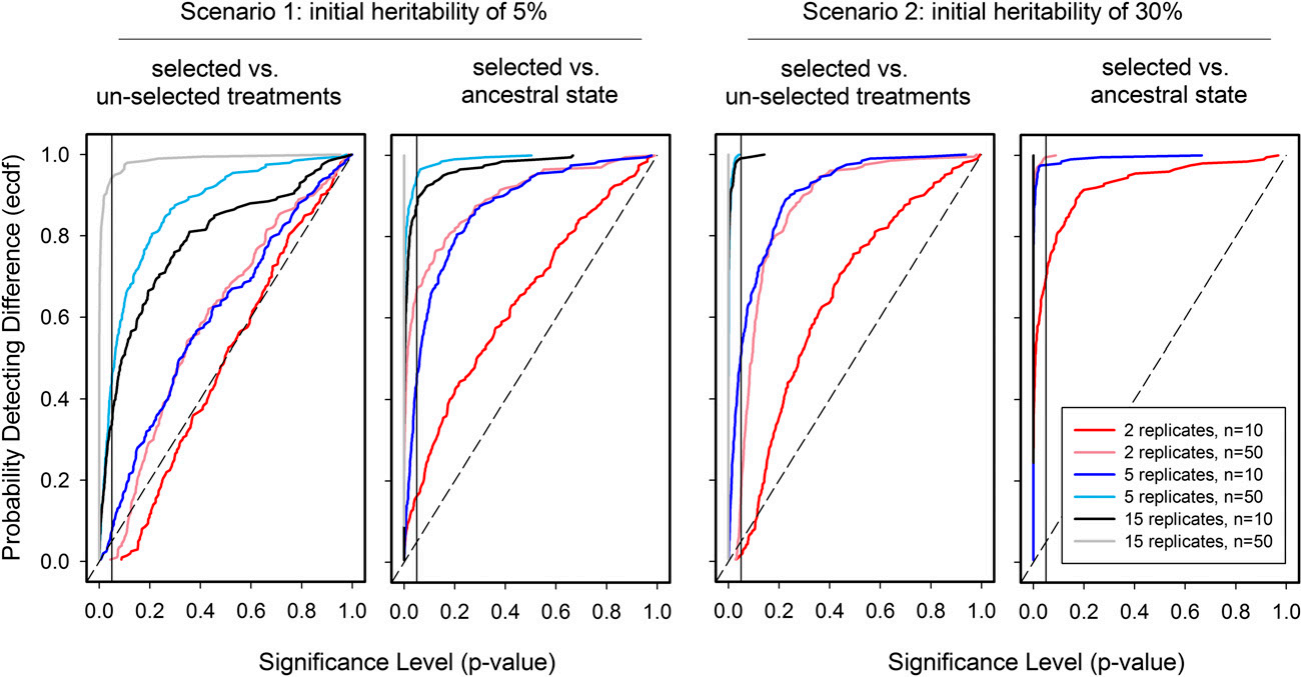
Detecting phenotypic differentiation and divergence. The statistical power to detect divergence or differentiation depends on many variables. Here, we consider how the number of replicate populations and sample size affect the power to detect responses to selection on a quantitative trait when reproduction occurs exclusively by selfing. We rely on the numerical simulation model presented in http://datadryad.org/, doi: 10.5061/dryad.bg08n; the reader can explore it to generate trait value trajectories and power curves as a function of standing and mutational genetic variance, population size and number of offspring, truncation and Gaussian selection, sample sizes, etc. The analysis we show is based on the simulated evolutionary trajectories of Figure 1. Individual fitness was defined as: *w* = exp{−[offspring_trait_value − optimum]^2/[2*(intensity^2)]} (Figure 1, right plot), with the new fixed phenotypic optimum set to ∼1 SD from the ancestral phenotypic distribution, and an intensity resulting in an initial linear selection gradient of 0.15. These are realistic numbers for natural populations (Kingsolver *et al.* 2001), but in the laboratory selection may be more substantial. The trait value of each hermaphrodite in the simulations is defined by its breeding value plus a stochastic component, assumed both to follow Gaussian distributions. Each hermaphrodite produced a fixed number of 10 offspring, with each one of them being represented in the next generation with a probability given by the fitness function, while keeping population size constant at 1000 individuals. This number was the approximately the effective population size of EE done by Teotónio *et al.* (2012), employing nonoverlapping and constant adult densities of 10^4^ (Chelo and Teotónio 2013). Many studies, however, employ overlapping generations, and do not test for the stability of age-structure during EE. Even though densities may be large in these studies, it is unclear which effective population sizes are realized (Box 1). For all plots, segregation/mutational heritability was of ∼0.15% at each generation, in line with *Caenorhabditis* MA estimates, *e.g.*, (Salomon *et al.* 2009). But note that trait heritability will change during EE depending on how selection and genetic drift influence standing levels of genetic variation. Once EE is done, testing for divergence or differentiation requires that replicate populations within each treatment show random effects due to genetic drift and uncontrolled selection. When employing frequentist statistical modeling, taking replicate populations as a fixed factor is incorrect since the degrees of freedom over which the significance of evolutionary responses are tested will be inflated. Using a linear mixed effects model, we tested for differentiation of unselected and selected populations after 50 generations by sampling 10 or 50 individuals from each of 2, 5, or 15 replicate populations. This model assumes that the heterogeneity among replicates is the same between treatments, which may not be true. Left panels show the probability of detecting differentiation (the cumulative density distribution of the simulations) as a function of significance level, for a trait that has a starting heritability of 5% (scenario 1 in Figure 1) or 30% (scenario 2 in Figure 1). Right panels show similar power curves when testing for divergence, where EE populations at generation 50 are compared to the ancestral state. The null hypothesis is that there is no divergence or differentiation (the identity dashed line). This analysis shows that detecting divergence is much easier than differentiation, and that a moderate to high number of replicate populations and high sample sizes are necessary to be confident that divergence or differentiation are due to selection (power of at least 80% at the significance level of 0.05; vertical line). Sampling several time points during EE will generally increase power, although it is often unclear how to model for trait autocorrelations across generations and if linear or nonlinear trajectories are expected (see Figure 1).

EE practitioners employ laboratory or field manipulations to understand the processes that lead to, and the mechanisms underlying patterns of, genetic and/or phenotypic diversity revealed by populations across multiple generations. The basic approach is straightforward, with most experiments being leveraged on the insights that can be provided by comparing phenotypes, and/or genotypes of populations evolved under experimenter-imposed conditions with those of an ancestor (divergence) or a control group (differentiation). Using numerical simulations, we show in Figure 1 how phenotypic responses would typically look like during EE, and in Figure 2 the statistical power to detect divergence or differentiation as a function of sample size and number of replicate populations. In Appendix 1 in File S1, we discuss some of the scaling and transformation problems in EE studies.

Because the development of evolutionary theory has far outpaced the generation of relevant data (genome-sequence data being a singular exception), using EE to confront long-standing problems has the potential to illuminate our understanding of evolution for decades to come provided a few qualifications are appreciated. The most important considerations are the potential difficulty in balancing simplicity and realism in contrived laboratory experiments (Huey and Rosenzweig 2009), the uncertainty surrounding the relevance of observations from EE for natural populations (matos *et al.* 2000), and current limitations on our ability to rapidly phenotype large numbers of individuals in the relevant environment—the one in which EE was performed and where fitness can be estimated. In principle, the power of EE studies derives from the fact that they are designed, repeatable experiments; in practice, experimental design must be carefully considered to avoid unintended consequences and alternative explanations that are impossible to distinguish from the hypothesis that the experimenter originally hoped to address. The general advice for beginning students is to limit the number of variables manipulated while controlling for laboratory domestication.

Doing EE well requires careful planning and organization, and the commitment to spend at least several hours transferring individuals every few days for months, or even years, on end. Why would anyone take on such a thing when they could start today on a comparative study in which nature has already done most of the work? Since students of evolution are primarily driven by “why” questions (mayr 1961), they will often be left wanting by results of comparative studies alone. Evolution is an inherently historical process, and so the vagaries of history are often expected to play an important role in determining evolutionary outcomes, and yet are likely to be completely opaque to us unless we can actually control and follow the structure of that history. While phylogenetic methods can be applied to infer the underlying evolutionary processes and ancestral character states (felsenstein 2004), this approach still involves a good deal of guesswork since very different processes can plausibly lead to the same evolutionary outcome (leroi *et al.* 1996; Sanderson and Hufford 1996). And while the comparative perspective is vital for revealing patterns of natural diversity and generating hypotheses about the causes of those patterns, we can, in principle, eliminate much of the guesswork by directly observing evolution in controlled settings.

### Advantages and limitations of Caenorhabditis for experimental evolution

Some early studies on thermal adaptation (brun 1966a,b,c; lower *et al.* 1968) and rates to lethal mutations (rosenbluth *et al.* 1983; clark *et al.* 1988) notwithstanding, application of EE methods with *Caenorhabditis* species did not begin in earnest until the 1990s with the work of johnson and colleagues on the evolution of aging (Johnson and Hutchinson 1993; Brooks *et al.* 1994; Walker *et al.* 2000; Jenkins *et al.* 2004), that of LaMunyon and Ward on sexual selection (laMunyon and ward 1995, 1997, 1998, 1999, 2002), and that of keightley and colleagues on mutation rates and their effects (keightley and Caballero 1997; davies *et al.* 1999; Vassilieva and Lynch 1999; Denver *et al.* 2000; Vassilieva *et al.* 2000; azevedo *et al.* 2002; Peters *et al.* 2003). There is now a remarkably broad range of problems being addressed with *Caenorhabditis* EE (Table 1, and see Gray and Cutter 2014).

One reason for the delay in adopting *Caenorhabditis* in EE is that natural intraspecific and interspecific diversity was little studied until the 2000s (phillips 2006; kiontke *et al.* 2011), at least in part because large-scale collection of natural isolates was not feasible until felix *et al.* (2014) discovered effective ways of targeting collections in natural habitats of rotting fruit and plant stems. This lack of knowledge limited the scope of questions that could be tackled. In particular, since most short-term evolution in sexual species is thought to occur from standing genetic variation rather than from new mutational variance (hill 1982; caballero and santiago 1995; matuszewski *et al.* 2015), the highly inbred and genetically depauperate *C. elegans* laboratory strains that were available at the time were initially perceived as being of little use for testing evolutionary theory.

We now understand that natural populations of *C. elegans* are inbred due to selfing, and that outcrossing is rare (Chasnov and Chow 2002; Stewart and Phillips 2002; Barriere and Felix 2005; teotónio *et al.* 2006). Selective sweeps and background selection appear to dominate its population genetics (Cutter and Payseur 2003; rockman *et al.* 2010; andersen *et al.* 2012; thomas *et al.* 2015), resulting in poor gene diversity and extremely strong linkage disequilibrium (graustein *et al.* 2002; sivasundar and Hey 2003; barriere and felix 2005; haber *et al.* 2005; cutter 2006; Thompson *et al.* 2015). Related outcrossing species display extensive natural variation, however (cutter *et al.* 2013; Dey *et al.* 2013; Cutter 2015), with *C. brenneri* being perhaps the most genetically diverse eukaryote described so far (Dey *et al.* 2013). And now, populations of *C. elegans* constructed via the hybridization of wild isolates have been developed for the specific purpose of studying evolution from standing genetic variation (Table S1 in File S1 lists a few of the resources for EE available in *C. elegans*).

Many of the same features that make the natural population genetics of *Caenorhabditis* somewhat odd also make it ideally suited for laboratory approaches to studying evolutionary questions (Frezal and Felix 2015; petersen *et al.* 2015). For example, questions about the interaction between reproduction system and standing genetic variation can be nicely matched with the choice of experimental design (*e.g.*, manipulating sex determination in *C. elegans* or using natural populations of the highly polymorphic *C. remanei* as experimental starting points). Of course, the real power here is the ability to capitalize on decades of hard work on the genetics and functional biology of *C. elegans* that has been provided by the broader *C. elegans* research community (corsi *et al.* 2015).

Because of these advantages, *Caenorhabditis* are now arguably the best metazoan models for the experimental study of evolution (Gray and Cutter 2014). Although worms cannot match the short generation times or large population sizes of microbes such as yeast or bacteria commonly employed for EE (bell 1997; kassen 2014), relatively long-term evolution is feasible with *C. elegans*; *e.g.*, lineages have been cultured under selfing for up to 400 generations (matsuba *et al.* 2012; katju *et al.* 2015), and maintained under different reproductive systems for up to 200 generations (theologidis *et al.* 2014).

Like many microbes, *Caenorhabditis* can be easily and reliably cryopreserved. Cryogenics allows one to halt evolution of ancestral stocks and permits accurate evaluation of repeatability and parallelism of evolution (denver *et al.* 2010; estes *et al.* 2011). Not only does the ability to cryopreserve stocks allow direct comparisons between evolved and ancestral states, it means that an experiment can live on after the initial period of evolution and new features of the experimental system can be investigated—a major advantage over other metazoan systems such as *Drosophila*.

But the feature of androdioecious *Caenorhabiditis* that is unique among metazoan experimental systems is that sex determination can be genetically manipulated, allowing researchers to obtain populations with variable ratios of males, females, and hermaphrodites (haag *et al.* 2002; baldi *et al.* 2009; beadell *et al.* 2011), and thus to achieve different degrees of selfing and outcrossing. This has allowed the role of segregation and recombination in evolution to be tested independently of confounding environmental factors (cutter 2005; morran *et al.* 2009b, 2011; baer *et al.* 2010; Chelo and Teotónio 2013; theologidis *et al.* 2014). In contrast, for microbes such as the yeast *Saccharomyces cerevisiae* (goddard *et al.* 2005; McDonald *et al.* 2016), and the green alga *Chlamydomonas reinhardtii* (colegrave 2002; Lachapelle and Bell 2012), or metazoans like the rotifer *Brachionus calyciflorus* (becks and Agrawal 2010; luijckx *et al.* 2017), manipulation of sexuality usually involves distinct life-histories imposed by different environments.

## Goals, Outcomes, and Interpretation of Experimental Evolution

### Domestication to laboratory conditions

When s. brenner decided to embark on a new research program based on studying a simple metazoan organism, he selected a *C. elegans* strain, N2, which had been propagated in the laboratory for perhaps thousands of generations (weber *et al.* 2010; sterken *et al.* 2015; Nigon and Felix 2016). Unbeknownst to him, this strain had already undergone extensive adaptation to laboratory conditions and probably became even more specifically adapted to what are now the standard *C. elegans* handling conditions (stiernagle 1999). It is now clear that the N2 strain is quite distinct from natural isolates of *C. elegans* for a variety of developmental, physiological, and behavioral traits. For example, specific alleles of *npr-1*, *glb-5*, and *nath-10* are only found in N2 and have wide-ranging effects (persson *et al.* 2009; mcGrath *et al.* 2011; duveau and felix 2012; andersen *et al.* 2014). Indeed, N2 is unique relative to the more than >200 wild strains whose genomes have been published thus far (andersen *et al.* 2012; cook *et al.* 2016), suggesting that certain traits, such as higher lifetime fecundity, lower ethanol tolerance, and lower propensity to aggregate, were unintentionally favored in laboratory environments involving short generation times, nonlimiting food and low-dimensional habitats.

A similar set of results has emerged from the analysis of another strain, LSJ2, originally derived from N2 and maintained in liquid culture for a period of 50 years (mcGrath *et al.* 2011). Adaptation to the high-density growth conditions experienced in liquid culture rendered individuals resistant to the pheromone-induced dauer larval formation observed in N2 and several wild *C. elegans* isolates—a response shown to result from deletions in two pheromone receptor genes: *srg-36* and *srg-37*. Comparison to a closely related *C. elegans* laboratory strain and an isolate of *C. briggsae*, both of which had experienced multiple generations in high-density liquid culture, revealed the same loss of resistance due to deletions in identical or a paralogous gene(s), respectively. This long-term laboratory selection has also modified allocation among N2’s life-history traits via modification of chromatin state by the NURF chromatin remodeling complex (large *et al.* 2016).

One important consequence of these results is that researchers who study the so-called “standard,” “wild-type,” or “reference” strain of *C. elegans* should recognize the possibility that the biological processes of interest could have been perturbed by adaptation to laboratory conditions. If nothing else, arguments regarding the putatively adaptive nature of a given discovery should be tempered until they can be verified with other natural isolates (cutter 2015; frezal and felix 2015; petersen *et al.* 2015). The genetic differentiation generated by long-term selection could be used to identify the genetic changes underlying the response to selection, yielding insights into the functional basis of the response. However, because these particular domestication studies were not specifically designed for this purpose, they cannot be used to test specific evolutionary hypotheses in the way that replicated and controlled designs can be. While the capacity for parallel adaptive response revealed by the study of McGrath *et al.* (2011) is intriguing, it is still unclear whether this process (not to mention the particular genes or alleles involved) is relevant to evolution beyond the laboratory.

Two additional aspects of laboratory domestication potentially confound the interpretation of responses to a given experimental treatment, especially when evolution occurs from standing genetic variation instead of mutational input. First, laboratory conditions are a novel environment to which only a few genotypes will, by chance, be either very well adapted or very maladapted (Service and Rose 1985). Second, for populations maintained even at relatively large sizes, inbreeding depression will generate positive genetic correlations among fitness components (rose 1984). An example of the latter problem is given by the study of carvalho *et al.* (2014) who conducted EE using the populations with standing genetic variation of teotónio *et al.* (2012) and found that, after 100 generations at stable and intermediate selfing rates, hermaphrodite early-life fertility and lifespan increased when they were selfed, but not when they were outcrossed. This result is seemingly at odds with theory on the evolution of aging, which predicts a fitness trade-off between early and late life-history (williams 1957; Hamilton 1966). However, inbreeding depression may in this case explain the positive genetic correlation between fitness components simply because more recessive alleles are expressed under selfing than outcrossing. Thus, care must be taken to avoid misinterpreting consequences of domestication as selective responses to applied experimental treatments.

### Natural selection and genetic drift

**Figure 3:**
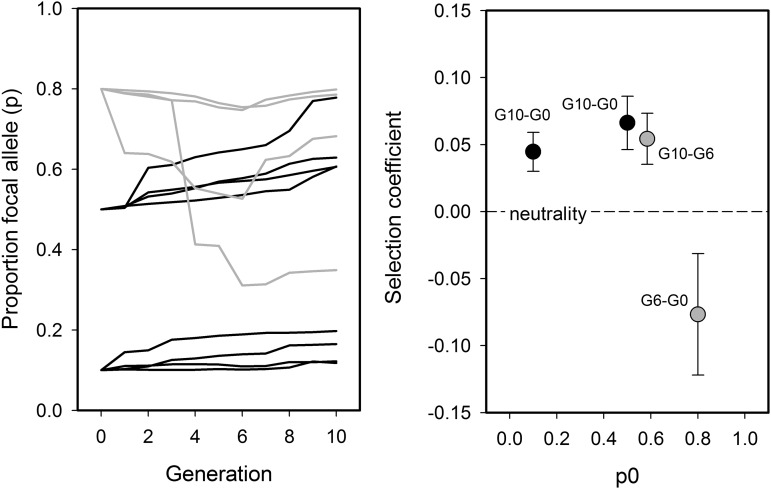
Measuring natural selection and genetic drift. Fitness can be estimated in *Caenorhabditis* EE with competitions between ancestral and derived populations with a “tester” strain that is distinct in morphology, for example by the expression of a green fluorescent protein (GFP) (Morran *et al.* 2009a; Theologidis *et al.* 2014). EE populations and tester are usually placed at equal frequencies, and their frequency is followed for one generation in the same environmental conditions as those during EE. Their relative proportion after the competition can then be taken as an estimate of relative fitness, assuming that there is no assortative mating. This way of estimating fitness is appropriate for populations cultured under nonoverlapping generations and stable densities. Fitness components can be measured in a similar fashion. For example, to estimate male fitness, EE males can compete with tester GFP males for the fertilization of tester females (these can be *fog* or *fem* mutants, for example), with male fitness being the relative offspring proportion of wild-type *vs.* tester GFPs (Teotónio *et al.* 2012). For *Caenorhabditis* EE with overlapping generations, and when a stable age-structure has been reached, then relative fitness can be estimated as the solution of Σ*e^−rx^ l*(*x*)*m*(*x*) *=* 1 for *r*, the “Malthusian” intrinsic growth rate parameter (Roff 2008). Here *l*(*x*) is the proportion of individuals surviving to age *x*, and *m*(*x*) the fecundity at age *x*, see *e.g.*, Estes *et al.* (2011) for an application of this model. For populations that have not reached stable age distribution *r* may be taken as absolute “Darwinian” fitness, by measuring the total number of offspring produced during an individual’s lifetime. In population genetics studies, the relative fitness of particular alleles at a given locus can be measured as the log ratio of their frequency change across generations, a coefficient that is usually called “selection coefficient”: *s* = {ln[*pG*/(1−*pG*)] − ln[*p0*/(1−*p0*)]}/*G*; where *pG* and *p0* are the frequencies of the allele in question after *G* generations, and at the beginning of the competition *G = 0*, respectively. Similar but more complicated expressions can be used when there is density- or frequency-dependent selection (Chevin 2011). In genome-wide studies, neutral polymorphisms (SNPs, for example), linked to the putative selected alleles, but unlinked to each other, can be employed to measure selection. The plots illustrate such examples: left, frequencies of the allele of interest; right, selection coefficients, with one SEM between the four replicates/neutral SNPs. Note that, with frequency-dependence, selection coefficients may change sign during the competitions (gray). The variance in allele frequency dynamics with EE can also be (retrospectively) used to estimate the effective population size, and thus the extent of genetic drift and inbreeding (Goldringer and Bataillon 2004; Chelo and Teotónio 2013). Typically, the effects of genetic drift at the genotype or phenotype levels are only accounted for as the random variation observed between replicate populations subject to the same treatment. One exception is when genetic drift can be thought of as a branching process and the growth rates of alternative types (be it alleles, genotypes, phenotypes) are independent. This occurs when mutants appear in very low numbers and invade a “resident” population composed of alternative types. Under discrete and nonoverlapping generations, with stable densities, and with successful offspring distributions following Poisson distributions, the proportion of invasions that are not successful is an estimate of the extent of genetic drift (Haldane 1927; Kimura 1957). Even for strongly selected mutants genetic drift can be estimated as the proportion of mutant invasions as: (1 – probability of extinction) = 1−e ^−2^*^sn^*^(^*^Ne^*^/^*^N^*^)^, where *s* is the selection coefficient, *n* the number of mutants invading, and *N_e_* and *N* the effective and census population size, respectively. These considerations are important since transitions in character states (for example between outcrossing and selfing) are ruled first by genetic drift, and only later by selection.

The central goal of evolutionary biology is to understand the evolution of adaptive traits—the organismal features that enhance survival and/or reproduction. *Caenorhabditis* offers an attractive model for performing rigorous experimental tests of theory describing rates of adaptation in terms of the fundamental processes of mutation, segregation, recombination, and natural selection. EE approaches can serve both as a source of potentially causal variants (*e.g.*, mutations captured from sequencing of experimentally evolved lines), and as a method for characterizing their phenotypic consequences and evolutionary dynamics (see Table 2 for types of EE approaches). The latter is usually achieved by measuring fitness, and fitness component, responses in the environment(s) where evolution took place. The appropriate measure of fitness, however, depends on whether generations are overlapping or nonoverlapping, if reproduction is continuous or discrete, if there is density-dependence, and if environments are temporally or spatially variable (roff 2008). While many of the parameters can be controlled by researchers in evolution experiments, some cannot, and fitness proxies must be found. A review of the conceptual underpinnings of defining fitness and fitness components in the context of EE would require a book-length treatise (mueller 2010; chevin 2011). We briefly review current EE methods for measuring natural selection and genetic drift in Figure 3, and, in Box 1, we expand about how population size may determine the efficiency of selection.

**Table 2:** Types of EE

Experiment type	Goal(s)	Design features	Output
Artificial selection	(i) Genetic architecture of specific trait(s), (ii) domestication	Experimenter defines fitness as a function of the value of the trait(s) of interest	Response to selection of the trait of interest; indirect responses of correlated traits
Laboratory natural selection	(i) Evolution of genetic systems, (ii) genetic architecture of the response to natural selection on fitness in a defined context	Experimenter imposes selective milieu; nature decides what the relevant traits are	The multivariate phenotype, genome-wide allele frequencies in SNPs, CNVs, etc.
Competition experiments	(i) Fitness of specific genotypes in a defined context, (ii) find the loci of adaptation	Experimenter defines starting frequencies of different identifiable genotypes, perhaps associated with phenotypes; nature selects among them	Derived allele (or genotype) frequencies, associated with phenotype frequencies
Reverse evolution	Test for nonadditive gene interactions: (i) Compensation of mutationally-degraded genotypes, (ii) natural selection erases history	Evolved population allowed to re-evolve under ancestral conditions	Some measure of phenotype or fitness
Invasion experiments	(i) Test for transitions in character state, (ii) measure genetic drift independently of selection	Rare genotypes introduced into a population; Highly replicated	Proportion of invasions that go extinct are observed
Inbreeding experiments	Dominance and epistasis as revealed by inbreeding and outbreeding depression	Inbred individuals (typically offspring of self-mating or sib-mating) are compared to outcrossed individuals	Some measure of phenotype or fitness, lineage survival during inbreeding
Mutation accumulation	Rate, spectrum, and distribution of mutational effects	Replicate populations derived from a known ancestor are maintained under minimal selection	Some measure of phenotype or fitness; molecular mutations measured by sequencing

Box 1 **Census and effective population sizes**

Population size *per se* is often a variable of interest in EE. For example, one might ask: Does trait X evolve qualitatively differently in large populations than it does in small populations? In such cases, the role of population size is unambiguous conceptually, but there are at least two potentially confounding empirical factors that must be taken into account: population density and the distinction between census size and effective size of the population. Population density is the number of individuals per unit resource, where the “resource” can be food, space, potential mates, etc. The particulars of *Caenorhabditis* biology add several complications where density is concerned. First, since the food source is usually live bacteria, the amount of food itself is not necessarily monotonically decreasing with time, nor is its quality constant in time. In some cases, relatively small differences in initial census size may lead to significant nonlinearity in the relationship between population size and starvation resistance over time (Artyukhin *et al.* 2013).

Effective population size, *N_e_*, can be defined as the size of an ideal population that experiences genetic drift and inbreeding to the same degree as the population of census size *N* in question (Wright 1969). Assuming an infinitesimal model of trait inheritance (meaning an “infinite” number of alleles each with “infinitely” small effects), differences in the intensity of selection (*i*; defined as the trait value difference between selected parents relative to the population mean) lead to predictable differences in *N_e_*: the greater *i* is, the smaller *N_e_* will be (Robertson 1960, 1961). This is because trait evolutionary change depends only on its covariance with fitness. From the perspective of EE, the important considerations regarding *N_e_* are: is the relationship *N_e_*/*N* consistent under a particular set of experimental conditions? And, does the relationship *N_e_/N* differ consistently between experimental conditions? If the answer to the first question is “no,” variation in evolutionary outcomes among replicates will be greater than if the answer is “yes”. If the answer to the second question is “yes,” it may be a real problem, because adjusting *N* among treatment groups such that the different treatments have equivalent *N_e_* may lead to meaningfully different experimental conditions, such as density, between different treatment groups.

If the effects of variation in population size *per se* are not of interest, population size will be a feature of the logistics of the experiment, chosen by the experimenter on practical grounds. Nevertheless, the choice of population size will have important implications. First, all else being equal, the smaller the population, the greater the influence of genetic drift and demographic stochasticity. Thus, the smaller the population, the greater the extent of replication needed to detect consistent evolutionary outcomes that may be due to selection. Second, the efficiency of selection is governed by the joint parameter *N_e_s*, so the smaller the experimental population, the larger the fitness effect of an allele must be in order to consistently contribute to a response to selection. Similarly, the smaller the population, the greater the influence of deleterious alleles on the outcome.

The classic population genetics model, called the Wright-Fisher model, is appropriate to describe genetic drift and inbreeding when generations are nonoverlapping, and reproduction is discrete in time. This is usually called the “neutral” model. Any deviation from expectations under this neutral model is interpreted as resulting from selection: directional, stabilizing or balancing, and disruptive. One should bear in mind, however, that in the presence of linked selection (hitch-hiking of neutral alleles with selected ones: “Hill-Robertson” effects), genotype-by-environment, and epistatic interactions, the usefulness of the concept of *N_e_* is controversial (*e.g.*, Gillespie 2001; Proulx and Adler 2010; Neher 2013). But ignoring these complications, and using the expected variance of SNP marker frequencies across time, it has been shown that *N_e_* is about an order of magnitude lower than *N* in *C. elegans* EE employing discrete and nonoverlapping generations, even with significant selfing (Chelo and Teotónio 2013).

The variance of the distributions of several fitness components is responsible for *N_e_* being generally lower than *N* (Crow and Kimura 1970), since, just by chance, individuals may not survive to reproduction or leave offspring. In outcrossing populations, variation in male and female mating success cannot be ignored, while in selfing *C. elegans* populations accounting for variation in hermaphrodite reproductive success should be taken into account. With overlapping generations, where individuals from different generations interact with each other and reproduction may be continuous in time, the variances of the distributions of fitness components change with density and life-history stage, usually decreasing *N_e_*. Quantifying genetic drift and inbreeding as a birth–death process is likely appropriate for this scenario of overlapping generations.

Competition experiments (CEs) are the best approach for measuring natural selection (Figure 3 and Table 2). CEs are a class of evolution experiments in which individuals of different identifiable genotypes are allowed to evolve in head-to-head competition with each other. Competition is implicit in most evolution experiments; however, CEs are distinct in that investigators control the starting frequencies of the different types and the “trait” of interest is the change in frequency of these types across generations. They are primarily employed to understand how selection determines the frequency dynamics of alternative alleles or genotypes that are of *a priori* interest (Appendix 2 in File S1). Promisingly, CEs are beginning to be used for gene discovery and understanding the genetic basis of adaptive traits in so-called “evolve and resequence” experiments (Weber *et al.* 2010; Long *et al.* 2015). For this purpose, in *C. elegans*, one can readily think of competing subsets of existing recombinant inbred panels (Table S1 in File S1).

A related form of CE is an “invasion experiment,” in which a focal genotype is tested for its ability to invade a resident population of alternative type(s) (Table 2). In one form of an invasion experiment, novel genotypes are introduced at low frequencies to test the evolutionary stability of the “resident” genotype, such as the ability of outcrossing populations to resist the invasion of a mutation that leads to self-reproduction (Theologidis *et al.* 2014; Slowinski *et al.* 2016) and vice-versa (Wegewitz *et al.* 2009). In invasion experiments, competition can also be maximally restricted so that the different genotypes have independent growth rate dynamics. By controlling the initial frequency of “invaders” and population sizes, such experiments can be used to test theoretical predictions regarding the role of genetic drift in determining evolutionary outcomes (Figure 3). For example, Chelo *et al.* (2013b) introduced a fixed number of mutants into wildtype *C. elegans* populations of 1000 individuals, finding that these new mutants were frequently lost even when adaptive, and, conversely, tended to linger within populations at frequencies higher than the deterministic expectation when they had deleterious effects, in keeping with the classical theory of stochastic population genetics (Haldane 1927; Kimura 1957).

When we think of a selection experiment, we usually imagine shifting a population to a new environment. We might call such approaches “forward EE.” Some of the most powerful evolutionary studies in *C. elegans* have taken the opposite approach—“reverse EE”—by initially perturbing the genetics of the population and then observing how they respond to their ancestral environment, or to environments that allow the recovery of ancestral states by selection. In this fashion, one can assess if and how selection overcomes evolutionary history (Estes and Teotónio 2009). For example, following ∼240 generations of accumulation of deleterious mutations under relaxed selection, populations of *C. elegans* can recover ancestral fitness within only 60 generations when selection is reimposed (Estes and Lynch 2003; Estes *et al.* 2011). Interestingly, the rate of recovery was strongly dependent on the specific combination of mutations present within the genetic background before recovery was initiated. Careful examination of one of the ancestral lineages via whole-genome sequencing in denver *et al.* (2010) suggested that the fitness recovery to ancestral levels resulted in part from the evolution of compensatory epistatic interactions, albeit with some caveats (Box 2).

Box 2 **Some caveats in EE**

The studies of S. Estes, D. Denver and colleagues (Estes and Lynch 2003; Denver *et al.* 2010; Estes *et al.* 2011) were useful in terms of revealing the capacity for rapid, repeatable responses to a population genetic environment at phenotypic and DNA sequence levels, but it is instructive to consider some limitations of this work, and how they may be overcome in future *Caenorhabditis* EE research. First, the authors’ inability to genetically isolate candidate mutations meant that they could not fully substantiate their claim that reverse evolution of fitness was due to compensatory mutation, or characterize the exact nature of any deleterious-adaptive mutational interactions. Second, the Denver *et al.* (2010) study could only detect single base pair changes in the nuclear genome; the contribution of other mutation types or mitochondrial DNA sequence changes to the evolutionary response was thus unknown. Although detecting genome rearrangements can still prove technically challenging, we can now readily survey other mutation types, like structural and copy-number variants (Farslow *et al.* 2015), and, with the high sequence coverage achievable for mitochondrial DNA, evaluate evolution of heteroplasmic mutations (*e.g.*, Wernick *et al.* 2016). Lastly, a shortcoming of Estes *et al.* (2011) study was that fitness of the evolved lines was quantified under standard, benign laboratory conditions rather than in the high-density, competitive conditions under which evolution had occurred; this left room for lingering questions regarding how genotype-by-environment, maternal, or other transgenerational effects may have contributed to the rapid response. A better approach would have been to assess fitness changes using competitive head-to-head assays wherein evolved lines are competed against a GFP-marked strain after culturing of populations in a “common garden” for a few generations (Figure 2 and Figure 3).

**Figure 4:**
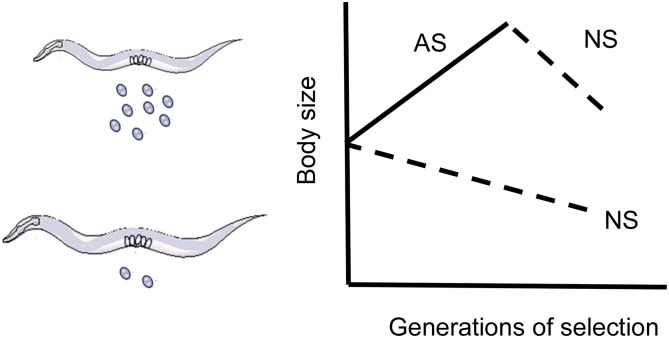
Artificial *vs.* natural selection. A good example of the use of artificial selection to test a specific genetic hypothesis is provided by the work of Azevedo *et al.* (2002), who selected for increased and decreased body size on replicated highly inbred (and presumably isogenic) populations of *C. elegans* of 48 generations. They were able to generate a rapid response to decreased body size but not to increased size. Because the populations were initiated without any standing variation, the response to selection must have been due to the contribution of novel mutations, indicating that the asymmetry in the response to selection is caused by an asymmetry in the distribution of mutational effects on body size—something that was verified via direct estimates of the mutational effect (Azevedo *et al.* 2002; Ostrow *et al.* 2007). These results suggest that either increasing body size is difficult because of a small set of genetic targets relative to decreasing body size, and/or increases in body size are constrained because of the pleiotropic effects of new mutations on other fitness components. The plots show figure illustrates a putative trade-off between body size and fecundity such that artificial selection (AS, line) for increased body size would lead to the fixation of deleterious mutations across replicate populations, as observed after stopping AS. Conversely, natural selection (NS, dashed lines) would result in a correlated decrease in body size. One important distinction between AS and NS is that the first usually involves “hard” truncation selection, where individuals below a certain trait value threshold do not contribute to the next generation.

Much of our understanding of the genetics of complex and quantitative traits comes from artificial selection experiments, particularly those whose aim is to improve plant yield and animal production in agriculture (Lynch and Walsh 1998). Artificial selection has also been commonly used in EE research, mainly to test hypotheses about the genetic basis of the trait under investigation and estimate quantitative genetic parameters such as heritabilities and genetic correlations. Artificial selection involves the intentional breeding of individuals with particular trait values, which may or may not have been otherwise favored by natural selection (Figure 4). The primary distinction here is that in artificial selection experiments, the investigator knows the target of selection, while in “natural selection in the laboratory” experiments, any feature of the organism that increases fitness within that environment is expected to increase in frequency.

### Mutation and standing genetic variation

The constant pressure of new mutations, most of which will be detrimental to their carriers, is a likely contributor to the origin and evolution of many biological features (sexual reproduction, genetic incompatibility, and genome architectures), and is thought by some to be the primary driver of long-term evolutionary patterns (Lynch *et al.* 2006; jones *et al.* 2007). Accurate measures of the rates, molecular spectra, and distributions of fitness effects of mutations are thus critical for many applications of evolutionary theory, including inferring evolutionary relationships, testing for selection on molecular sequence, estimating effective population size from standing levels of neutral genetic variation, and parameterizing population genetic models (Lynch *et al.* 1999).

**Figure 5:**
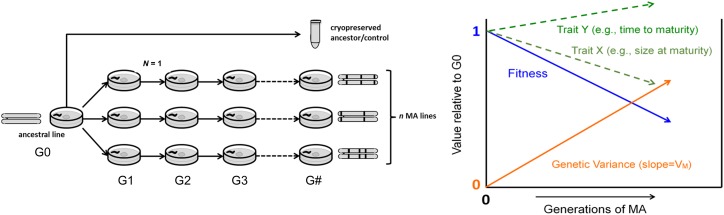
Mutation accumulation (MA) experiments. Left, illustration of how an MA experiment is performed, adapted from Halligan and Keightley (2009) and Katju *et al.* (2015). *G* indicates the generation of EE, *N* the number of individuals allowed to reproduce within each MA line, and *n* the number of MA lines. We schematize a diploid chromosome devoid of genetic variation in the ancestor that accumulates and fixes different (independent) mutations within each MA line. The cryopreserved ancestor and/or control is usually phenotyped alongside the MA lines. Right, graphical representation of expected outcome of an MA experiment. The blue line shows the expected trajectory of evolution of fitness among MA lines, which is unknowable in practice. Since most mutations will be deleterious in a well-adapted population, it is expected that fitness will decline with MA. Trait X represents the expected average evolutionary trajectory of a trait positively correlated with fitness; size at maturity is a typical example. Trait Y represents a trait negatively correlated with fitness; time to maturity is a typical example. The orange line represents the genetic variance between the MA lines, which (ideally) is zero in the G0 common ancestor of the MA line. The per-generation increase in the genetic variance is the “mutational variance,” *V_M_*.

To accurately characterize the mutational process, an unbiased sample of spontaneous mutations is needed; however, the standing allelic variation is a biased sample because it has been previously screened by selection. What is needed is a way to neutralize selection to the maximum extent possible so that new mutants, despite their fitness effects, can be fixed within replicate populations (Figure 5). This is the basic principle underlying the method of “mutation-accumulation” (MA). An MA experiment is, in essence, EE stood on its head: instead of experimentally investigating what evolves when a particular selective regime is imposed, the question of interest is: what evolves when selection is removed? The basic principles of MA are outlined in Appendix 3 in File S1, as are some general findings about the mutational process in *Caenorhabditis*.

Although mutation is crucial for long-term evolution, in sexually reproducing organisms, short-term evolution on the order of tens of generations mostly occurs from standing genetic variation. Explicit tests of theoretical expectations regarding the relative timeframes where mutation and/or standing genetic variation are important are mostly missing, however, not only in *Caenorhabditis* but also in other model organisms.

Evolution from standing genetic variation can occur through the sorting of extant genotypes by segregation or through a combination of segregation and recombination between existing genotypes. For *Caenorhabditis* EE, the population genetic consequences of selfing *vs.* outcrossing should be considered as breeding mode is expected to influence the degree of effective segregation and recombination (Appendix 4 in File S1). In general, selfing reduces effective population sizes, particularly if background selection of deleterious recessive alleles is present, potentiating genetic drift and the loss of neutral diversity. When considering multiple loci, however, neutral diversity can be maintained under selfing by hitchhiking with (positive) selected alleles and/or because of “identity disequilibrium” generated by genotypic correlations. Understanding selection under selfing is highly difficult but we do know that it fundamentally depends on the degree of dominance within loci and epistasis between loci.

To address the problems of evolution from standing genetic variation, EE with *C. remanei* and *C. elegans* have employed hybrid ancestors constructed from crosses of several wild isolates (LaMunyon and Ward 2002; Anderson *et al.* 2010; Schulte *et al.* 2010; Chen and Maklakov 2012; Teotónio *et al.* 2012; Sikkink *et al.* 2014b; Palopoli *et al.* 2015). The dioecious *C. remanei* is highly polymorphic, with an average per locus nucleotide diversity on the order of 5% (Cutter *et al.* 2006), and, despite the potential for outbreeding depression during crosses among these isolates (Dolgin *et al.* 2007), the resulting hybrid strains retain plenty of genetic variation available for selection during EE (Chen and Maklakov 2012; Sikkink *et al.* 2014b, 2015). The same is true for *C. elegans* hybrid ancestors (Anderson *et al.* 2011; Masri *et al.* 2013; Carvalho *et al.* 2014), despite displaying average polymorphism levels of only 0.2% (Cutter 2006). Recently, however, sequencing of the Hawaiian CB4856 strain genome revealed that polymorphism in *C. elegans* can be quite high, reaching 16% and possibly higher in some genomic regions (Thompson *et al.* 2015). It is perhaps not surprising then that EE responses in *C. elegans* starting from hybrid populations are comparable to those of obligate outcrossing species. Nonetheless, these *C. elegans* hybrid populations have been shown to lose much of their initial polymorphism via purging due to outbreeding depression during strain construction (Teotónio *et al.* 2012; Chelo *et al.* 2013a).

It is common to consider the evolutionary response of individual phenotypic traits (*e.g.*, “heat tolerance”) to selection, but organisms are, of course, not merely collections of atomized traits. Multivariate evolutionary responses will depend on how pleiotropy, linkage disequilibrium, and inbreeding/assortative mating have shaped genetic variances and covariances among traits [summarized in the genetic variance-covariance matrix **G**; (Lande 1980; Phillips and McGuigan 2006)]. Because of its highly selfing sexual system, resulting in strong linkage and inbreeding, multivariate evolution is expected to be more constrained in *C. elegans* than in *C. remanei*. In the hybrid *C. elegans* populations of Teotónio *et al.* (2012), 100 generations of EE under 50% of partial selfing or 100% of obligate outcrossing similarly reduced the initially high linkage disequilibrium to background levels for genetic distances ∼1 cM (on a F2 map scale, where each chromosome is 50 cM). Most evolution appeared to result from single-locus selection with few signs of reduced heterozygosity signaling large-scale sweeps (Chelo and Teotónio 2013). But, depending on chromosomal location, and because recombination rates are not monotonic along the chromosomes (Rockman and Kruglyak 2009), this small amount of linkage still means that hundreds to possibly thousands of alleles at different loci will tend to be inherited together, and have the potential to generate significant genetic correlations among traits.

Just as the fitness effects of new mutations can be characterized by means of an MA experiment, the fitness effects of standing genetic variants can be assessed by means of an “inbreeding experiment.” As with MA, several lineages are derived by selfing or extreme inbreeding with the goal of reducing heterozygosity and fixing alternative alleles within each lineage in a neutral fashion. A reduction of fitness among inbred lineages relative to the ancestral outbred population indicates inbreeding depression, and deviations from expected levels of homozygosity at marker (neutral) loci can reveal if inbreeding depression is due to strongly deleterious recessive alleles that are purged during the inbreeding experiment (Dolgin *et al.* 2007; Ebel and Phillips 2016). In contrast, deleterious recessive alleles in repulsion linkage disequilibrium (“associative overdominance”) or truly overdominant alleles can be maintained through balancing selection during inbreeding (Mukai *et al.* 1964; Ohta and Kimura 1970; Ohta 1971), and provide an alternative source of inbreeding depression. Distinguishing between these two latter alternatives is, however, notoriously difficult (chelo *et al.* 2013a).

### Data availability

R code for simulation of EE is deposited at http://datadryad.org/ doi: 10.5061/dryad.bg08n.

## Exemplars of Caenorhabditis Experimental Evolution

### Maintenance of genetic variation

One of the most enduring questions in evolutionary biology is: *Why so much genetic variation?* The discussion surrounding that question will be familiar to most readers of *GENETICS*; it suffices to say that the poles of the issue are “Mutation + Drift” at one end, and “Balancing Selection” at the other. In recent decades, the debate has centered on variation at the molecular level, but arguments long antedates the molecular era. (Dobzhansky 1937; Lewontin 1974; Kimura 1983; Charlesworth 2015).

With respect to the understanding of molecular variation, EE, including in *C. elegans*, has made a critical contribution by providing (nearly) unbiased estimates of the rate and spectrum of spontaneous mutation unencumbered by selection (Appendix 3 in File S1). Direct estimates of the per-nucleotide, per-generation mutation rate, *µ*, in model organisms provide a critical reality-check on indirect estimates of *µ* from sites putatively free from natural selection (*e.g.*, processed pseudogenes) because many tests of non-neutral molecular evolution assume there is a class of neutrally evolving sites that can serve as a reference (Kondrashov and Crow 1993; Cutter and Payseur 2003; Witherspoon and Robertson 2003). Direct estimates of *µ* are usually within a few-fold close to inferences drawn indirectly from putatively neutral sites, although there are occasionally incongruities between direct and indirect estimates, notably a consistently twofold higher direct estimate in humans (Shendure and Akey 2015).

The question “Why so *much*...” takes an interesting turn in *C. elegans*, into “Why so *little* genetic variation?”. Andersen *et al.* (2012) reported a large-scale survey of genome-wide nucleotide variation in *C. elegans*, and concluded that large regions of the genome must coalesce within a few hundred generations, presumably the result of one or more recent, global selective sweep. However, Thompson *et al.* (2015) compared the genomes of N2 and CB4856 (“Hawaii”), and reported that 2–3% of the genome appeared to harbor ancient segregating variation (“ancient” meaning an estimated average time of divergence on the order of 10^6^ generations), which they attributed to long-term balancing selection. Taken at face value, these two studies lead to the seemingly odd situation of most of the genome being genetically depauperate, but a dispersed fraction carrying ancient polymorphisms.

The question of *Why so much genetic variation?* applies equally to emergent phenotypic traits. If mutation and random genetic drift are the only forces at work, at mutation-drift equilibrium the standing genetic variance for the trait should equal *V_G_* *=* 2*N_e_V_M_* (Lynch and Hill 1986), where *N_e_* is the effective population size (Box 1) and *V_M_* the mutational variance (Appendix 1 in File S1). If an estimate of *N_e_* is available, as it would be from segregating nucleotide variation and an independent estimate of the per-nucleotide mutation rate (because *θ* = 4*N_e_µ*), a value of *V_G_* << *V_M_* implies the trait is subject to purifying selection and can be used as an approximate estimate of the strength of selection acting against mutations that affect the trait (Barton 1990). For example, Denver *et al.* (2005) compared *V_M_* and *V_G_* for hundreds of gene transcripts in four MA lines and five wild isolates of *C. elegans* (including N2). Small sample sizes notwithstanding, the results were impressive: of ∼3700 genes investigated, not one had a *V_M_*/*V_G_* ratio greater than the neutral expectation, from which it could be concluded that transcription is under strong and ubiquitous stabilizing selection.

More recently, this approach has been used to assess pattern of variation that are expected *a priori* to be under different types of selection (Salomon *et al.* 2009; Braendle *et al.* 2010; Etienne *et al.* 2015; Farhadifar *et al.* 2015). The results are summarized in Figure 3 of Farhadifar *et al.* (2016). Three salient results emerge. First, across a broad spectrum of traits, *V_M_* explains a large fraction of the variance in *V_G_* (∼90%), and the ratio *V_M_*/*V_G_* is well below the neutral expectation. That finding is entirely consistent with genetic variation in *C. elegans* being largely modulated by mutation and purifying selection (Rockman *et al.* 2010), but it also suggests that the strength of purifying selection must be quite similar among disparate traits. Second, for a large fraction of the traits, *V_G_* ≈ 500*V_M_*. These findings are also consistent with the conclusion of Andersen *et al.* (2012) that a large fraction of the *C. elegans* genome coalesces on the order of a few hundred generations, and they further suggest that random background selection across a highly linked genome predominates over trait-specific effects. However, *V_M_*/*V_G_* for some traits is clearly well below the trend, and the pattern is not random: life history traits and vulva development traits (Braendle *et al.* 2010) clearly experience stronger purifying selection than other traits.

The second class of unsupervised tests of non-neutral evolution involves the comparison of the between-population component of variance at putatively neutral marker loci (F_ST_), with the between-population component of genetic variance of phenotypic traits (Q_ST_) (Lande 1992; Spitze 1993). For traits experiencing significant stabilizing selection, Q_ST_ is predicted to be <F_ST_, whereas, for traits experiencing diversifying directional selection (*i.e.*, local adaptation), Q_ST_ is predicted to be >F_ST_. To our knowledge, this type of test has yet to be applied in *Caenorhabditis*. *C. elegans* is probably not conducive to Q_ST_/F_ST_ comparisons due to its unusual population structure, but *C. briggsae*, with its hierarchical population structure (Thomas *et al.* 2015), would seem to be an ideal candidate.

Although comparisons of *V_G_* and *V_M_* have not turned up compelling evidence for balancing selection, several lines of evidence support the conjecture that balancing selection may play a role in maintaining genetic variation in natural *C. elegans* (Thompson *et al.* 2015). For example, Greene *et al.* (2016) recently presented evidence that balancing selection on foraging behavior, mediated by chemoreceptor genes involved in pheromone signaling, has maintained genetic variation in foraging behavior, and, further, that the genomic region involved was one identified by Thompson *et al.* (2015) as a putative candidate for balancing selection. Competition experiments further revealed that the fitness effects depended on environmental context, such that one allele was favored in a constant-food environment, and the other favored in a patchy-food environment. In another example, Peters *et al.* (2003) characterized the fitness effects of EMS-induced mutations and found that 10/19 lines had point-estimates of fitness greater than the unmutated control, and that 3/19 lines were significantly overdominant; see also Manoel *et al.* (2007). Other examples of specific genes or gene complexes being potentially maintained by balancing selection have been found in natural *C. elegans* (*e.g.*, Gloria-Soria and Azevedo 2008; Seidel *et al.* 2008; Ghosh *et al.* 2012; Ashe *et al.* 2013), and, during EE from standing genetic variation, balancing selection has been invoked to explain maintenance of excess heterozygosity (Chelo and Teotónio 2013) and frequency-dependence (Chelo *et al.* 2013b). In addition, for outcrossing species, heterozygosity levels may have been underestimated (Barriere *et al.* 2009). Taken together, all these findings have important implications, because even a small number of overdominant loci (or recessive alleles at multiple loci in repulsion linkage) capable of generating balancing selection can have an outsize effect on the total inbreeding load (Dobzhansky 1955).

### Evolution of reproductive mode

Transitions from outcrossing to selfing are believed to be common in both animals and plants (Jarne and Auld 2006; Barrett 2008), and, in the particular case of *Caenorhabditis*, three independent transitions from dioecy to androdioecy have occurred (Kiontke *et al.* 2004). Even though androdioecy is a relatively rare reproductive system, and *Caenorhabditis* hermaphrodites somewhat unusual in their inability to outcross with each other, EE with *Caenorhabditis* has contributed to our understanding of selection during transitions from outcrossing to selfing, as well as the maintenance of mixed breeding modes during evolution.

Provided that mutation to self-compatibility is not limiting (Hill *et al.* 2006; Katju *et al.* 2008; Baldi *et al.* 2009), one route to selfing involves restrictions on outcrossing between males and females (*e.g.*, due to time required to find a mate). Hermaphrodites able to self autonomously will in this case be favored because they provide reproductive assurance and guarantee population survival (Lloyd 1976; Busch and Delph 2012). Second, selfing lineages may outcompete outcrossing lineages if the latter suffer from a “cost of males,” since males do not reproduce by themselves, but still consume ecological or developmental resources (Maynard Smith 1978; Lively and Lloyd 1990). A final route to selfing involves density-dependent selection among subpopulations (demes) for higher dispersal, which can be correlated with the ability to self and establish viable colonies without partners (Cheptou 2012).

Theologidis *et al.* (2014) took advantage of genetic manipulation of sex determination in *C. elegans* to show that the benefit of reproductive assurance is sufficient to explain transitions to selfing. As expected under this hypothesis, the successful invasion of hermaphrodites in male–female dioecious populations resulted in adaptation to a novel environment (high salt concentration) where outcrossing was restricted. Adaptation was not due to the loss of males, but rather to the replacement of females by hermaphrodites, as shown by the lower adaptive rates of androdioecious populations, which similarly lost males and reproduced exclusively by selfing by the end of EE. (Theologidis *et al.* 2014) did not test for density-dependent selection for dispersal among demes during transitions to selfing, but it is unlikely this form of selection favors selfing over outcrossing in natural *Caenorhabditis*. Not only are *C. elegans* males are more vagile than hermaphrodites (Lipton *et al.* 2004), they also have higher survivorship than hermaphrodites at the dauer stage (Morran *et al.* 2009a)—presumed to be the stage at which most dispersal occurs. The jury is nonetheless still out, and tests of the role of density-dependent selection on transitions to selfing are needed.

Because outcrossing in androdioecious *Caenorhabditis* is necessarily linked to the presence of males, transitions to selfing will be more difficult if male fitness components evolve prior to females being replaced by hermaphrodites—see Theologidis *et al.* (2014) for such an example. Similarly, because outcrossing allows for the continued generation of adaptive genotypes not easily accessible through selfing, any factor that reduces effective recombination may reduce the likelihood of the transition to selfing. The latter hypothesis was recently supported by the *C. elegans* EE study of Slowinski *et al.* (2016). In this example, in which populations evolved under ever-changing environmental conditions and fluctuating selection (in the form of a coevolving pathogen), hermaphrodites were unable to invade dioecious populations. In contrast, hermaphrodites readily invaded and reached high frequencies in populations evolved under constant environmental and selective conditions, either because they provided reproductive assurance, or because populations no longer paid a cost of producing males.

Once the transition from outcrossing to selfing has been achieved, classical theory suggests that the degree of standing inbreeding depression generated by deleterious recessive alleles will determine whether selfing persists or the population reverts to outcrossing (*e.g.*, Lande and Schemske 1985). Given sufficient time, selfing populations are expected to suffer less inbreeding depression than outcrossing populations since deleterious recessive alleles will be more effectively purged by selection. EE results with *C. elegans* are remarkably consistent with this hypothesis. Although inbred *C. elegans* populations with experimentally elevated mutation rates (*e.g.*, DNA-repair deficiencies or mutagenesis) do not maintain males (Cutter 2005; Manoel *et al.* 2007), whenever there is opportunity for the build-up of inbreeding depression because of high mutation rates, males are clearly favored and outcrossing can be maintained at higher rates than in unmutagenized controls (Morran *et al.* 2009b). And, as expected from theory, comparative evidence between selfing and outcrossing *Caenorhabditis* species indicates that male–female populations, such as those of *C. remanei*, maintain higher deleterious loads than predominantly selfing populations, such as those of *C. elegans* (Dolgin *et al.* 2007). The EE results from Chelo *et al.* (2013a) similarly suggest that partially selfing populations can maintain lower deleterious recessive loads than obligately outcrossing populations.

Not surprisingly, whenever there is standing genetic variation for fitness components related to outcrossing, like male reproductive success (LaMunyon and Ward 1998; Teotónio *et al.* 2006; Wegewitz *et al.* 2008; Murray *et al.* 2011), adaptation to novel environments is correlated with the evolution of higher outcrossing rates (Morran *et al.* 2009a, 2011; Teotónio *et al.* 2012; Slowinski *et al.* 2016). Adaptation is faster in obligate outcrossing populations than in facultative selfing populations, which in turn evolve faster than obligate selfing populations (Morran *et al.* 2009b). These observations are consistent with (i) balancing selection on overdominant loci favoring outcrossing (since more heterozygotes are produced by outcrossing than selfing); and/or (ii) outcrossing increasing effective recombination, and therefore generating more adaptive genotypes than selfing. Both of these scenarios have received experimental support. In the experiments of Teotónio *et al.* (2012), more heterozygosity was maintained than that expected by genetic drift and associative overdominance of linked deleterious partially recessive alleles (Chelo and Teotónio 2013), suggesting that outcrossing was favored because of balancing selection on overdominant loci. Further, consistent with multi-locus theory (Navarro and Barton 2002). Chelo and Teotónio (2013) inferred the presence of (negative) epistatic selection from the observed diminishing returns of fitness with increasing heterozygosity. Intriguingly, this form of epistasis can in part explain the evolution of recombination modifiers under partial selfing (Roze and Lenormand 2005), although no EE study has yet been undertaken to provide direct support for such an idea.

Regarding the second scenario, Morran *et al.* (2011) showed that increased outcrossing rates were only maintained during evolution in a fluctuating novel environment [as in (Slowinski *et al.* 2016), in the form of a coevolving pathogen], but not during evolution in a novel but constant environment in which case outcrossing rates increased initially and then decreased soon thereafter. Although rapid fitness recovery from mutationally degraded backgrounds can be achieved by selfing alone (Estes *et al.* 2004; Morran *et al.* 2010), these results strongly suggest that outcrossing increases effective recombination and allows the generation of more adaptive genotypes than does selfing.

Over time, selfing can lead to the evolution of epistatic gene complexes (by local adaptation or genetic drift) whose break-up by outcrossing will lead to outbreeding depression. Comparison of crosses between wild isolates in several *Caenorhabditis* species, followed by inbreeding experiments have indicated that the break-up of coevolved gene complexes could explain why selfing predominates over outcrossing in nature (Dolgin *et al.* 2007; Seidel *et al.* 2008; Gaertner *et al.* 2012; Chelo *et al.* 2013a; Gimond *et al.* 2013). Vexingly, the conundrum that outcrossing in *C. elegans* appears to be quite rare in nature but is not particularly difficult to maintain in the laboratory remains unresolved (Chasnov and Chow 2002; Stewart and Phillips 2002; Anderson *et al.* 2010). A complex balance between different forms of selection operating at different levels may explain the maintenance of partial selfing under a variety of conditions, some of which have been explored with EE, including: the evolution of sexual conflict between males and hermaphrodites (Chasnov 2013; Carvalho *et al.* 2014; Palopoli *et al.* 2015), the allocation of resources toward self-spermatogenesis or oogenesis in hermaphrodites (Anderson *et al.* 2010; Murray and Cutter 2011; Poullet *et al.* 2016), rapid adaptation to specialized environments (Morran *et al.* 2009a; Masri *et al.* 2013; Slowinski *et al.* 2016), and/or unresolved inbreeding depression (Chelo *et al.* 2013a). The topic of evolution of breeding modes will surely keep *Caenorhabditis* EE researchers occupied for many years to come.

### Evolution in variable environments

The difference in trait values displayed by a single genotype across multiple environments is known as phenotypic plasticity, long thought to be a key element in structuring both the response to selection in variable environments, and in the evolution of developmental and physiological systems (Via *et al.* 1995; Ghalambor *et al.* 2007). Upon encountering a new environment, population survival will depend on plastic genotypes closely matching the “optimum” phenotype (Price *et al.* 2003; Chevin *et al.* 2010). But, because the optimum phenotype can be matched by existing genotypes, the strength of directional selection is weakened and adaptation possibly hampered. Even if plasticity obviates the need for short-term adaptation, there may still be opportunity for selection of genotypes that improve performance in the novel environment, particularly if there is a cost associated with maintaining the developmental and physiological programs underlying phenotypes that are only rarely expressed. Initial plasticity in this case will facilitate long-term adaptation by allowing time for the “genetic assimilation” of the optimum phenotype in the novel environment by novel mutational or segregation/recombination input. The study of Sikkink *et al.* (2014b) in *C. remanei* has provided convincing evidence for such genetic assimilation, in line with classic work in *Drosophila* (Waddington 1953). Sikkink and colleagues adapted large, polymorphic populations to environments generating high levels of temperature and oxidative stress and observed rapid and fairly independent responses to selection alongside diminished plasticity when evolved populations were returned to their ancestral environments. The role that stochastic trait variation may play in genetic assimilation and in the evolution of trait “robustness” is currently untested (Felix and Barkoulas 2015). However, several *C. elegans* studies have found significant heritability for stochastic traits that could be under selection (Braendle and Felix 2008; Braendle *et al.* 2010; Duveau and Felix 2012).

A particularly fascinating form of plasticity results from the transmission of environmental effects experienced by a parent to the phenotype expressed by the offspring, *i.e.*, transgenerational plasticity. When developing individuals do not have the possibility to assess the environment they will face at the time of reproduction, phenotypic plasticity has little opportunity to be selected. Instead, and as long as there is an environmental correlation between generations, it is expected that mothers will provision, or cue, their offspring accordingly. Maternal effects could thus be especially important for adaptation to fluctuating environments. Dey *et al.* (2016) explicitly evaluated this idea by exposing *C. elegans* populations to either regularly or irregularly fluctuating normoxia–anoxia larval hatching environments. They observed the evolution of anticipatory maternal effects such that hermaphrodites were able to shift glycogen provisioning to developing embryos to achieve levels appropriate for the environment experienced by their broods in the next generation. Contrary to theoretical predictions (Proulx and Teotónio 2017), however, populations experiencing irregularly fluctuating environments failed to evolve an anticipatory response in the form of maternal “bet-hedging,” in which mothers randomize offspring phenotypes. Instead, evolution of longer-term (>2 generations) transgenerational effects may have been selected and promoted adaptation to fluctuating environments. Whether such long-term transgenerational effects are necessarily adaptive is still controversial (Wolf *et al.* 1998; Badyaev and Uller 2009; Uller *et al.* 2013; Burgess and Marshall 2014). Within *C. elegans*, it is increasingly apparent, however, that heritable nongenetic carry-over effects can persist for long periods—up to 30 generations or more (Katz *et al.* 2009; Burton *et al.* 2011; Luteijn *et al.* 2012; Shirayama *et al.* 2012; Ashe *et al.* 2013; Rankin 2015).

**Figure 6:**
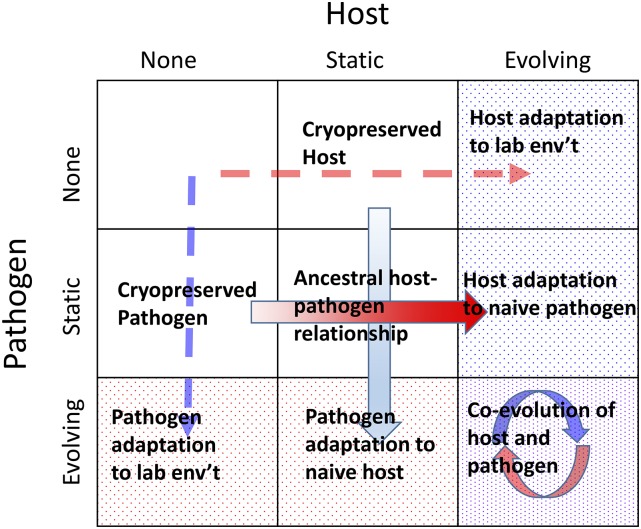
Host–pathogen coevolution experiments. Here we show a schematic relationship of experimental treatments in a comprehensive host–pathogen coevolution experiment. Columns show host treatments, rows show pathogen treatments. Cells with shading represent evolving experimental populations; arrows represent the direction of evolutionary causation.

Besides variable abiotic environments, interactions between host organisms and their pathogens and/or parasites are expected to generate strong fluctuating selection. Of special interest is the extent to which host–pathogen relationships are genotype-specific, and the ways in which evolution with coevolving pathogens (or hosts) differs from evolution in the presence of nonevolving pathogens (or hosts) (Box 3 and Figure 6). Two studies exemplify advances in our understanding of host–pathogen coevolution with *C. elegans* EE. In one, schulte and colleagues allowed 20 replicate populations of *C. elegans* to coevolve with the bacterial pathogen *Bacillus thuringiensis* (*Bt*) for 48 generations (Schulte *et al.* 2010). As controls, 10 replicates of the *Bt* pathogen, and 20 replicate populations of *C. elegans* were allowed to evolve in parallel in the absence of the other. As expected, the coevolved pathogen evolved increased virulence and the coevolved host evolved increased resistance when compared to the non-coevolved controls. Also as expected, the coevolved traits came with a fitness cost: several life-history traits (presumably correlated with relative fitness) decreased in the coevolved host and pathogen populations compared to the non-coevolved controls.

Box 3 **Host–pathogen coevolution experiments**

Signature features of coevolution are that the fitness effects of alleles in one (host or pathogen) species may depend on genotype frequencies in the other species, and that the effects of alleles in coevolved host populations confer high(er) fitness in the presence of the coevolved pathogen genotype. A straightforward prediction is that some salient property(s) of coevolved hosts and pathogens will differ from hosts evolved in the presence of non-coevolving pathogens, and vice versa, if coevolution has played an important role in the evolution of either partner. To unambiguously demonstrate such a difference, hosts must be evolved in the presence of coevolving pathogens, *and* in the presence of non-coevolving pathogens, and, similarly, pathogens must be evolved in the presence of coevolving hosts *and* with non-coevolving hosts (Figure 6), and the relevant properties compared among these groups and with the nonevolved ancestors. A comprehensive experiment would have five treatment groups: (1) Pathogens evolving to the laboratory environment in the absence of hosts (this would not be possible with an obligate parasite such as a virus); (2) pathogens evolving with nonevolving hosts (this can be easily done by serially passaging pathogens onto populations of naive hosts); (3) hosts evolving in the absence of pathogens (as in 1); (4) hosts evolving with nonevolving pathogens (as in 2); and (5) coevolving hosts and pathogens. Nonevolved cryopreserved ancestral pathogens, and hosts, would constitute the baseline control for such an experiment.

*Caenorhabditis* provide a uniquely powerful model system with which to experimentally investigate coevolution because they are so readily cryopreserved. *C. elegans* can host a wide spectrum of bacteria, fungi, protozoa, and viruses, as well as combinations thereof (*e.g.*, Dirksen *et al.* 2016). Moreover, unlike dipterans, which fly, and plants, which attract pollinators and herbivores that fly, nematodes carrying pathogens can be safely contained with a minimum of effort and expense. Those factors have made *C. elegans* a popular model system for characterizing the genetic basis of pathogen resistance and avoidance. Many studies have focused on specific mutants on the N2 background (*e.g.*, Mahajan-Miklos *et al.* 1999; Aballay and Ausubel 2001; Garsin *et al.* 2003; Troemel *et al.* 2006), but a number of studies have employed standard quantitative genetic line-cross analysis between N2 and the Hawaiian strain CB4856 to dissect the genetic basis of pathogen-related traits (Reddy *et al.* 2009; Andersen *et al.* 2014; Glater *et al.* 2014; Nakad *et al.* 2016). The latter have revealed significant genetic variation for a variety of pathogen-related traits (*e.g.*, olfactory behavior, oxygen avoidance, longevity, and innate immunity), often revealing genes that explain a significant fraction of the genetic variation. Most investigations of host–pathogen interactions involving *C. elegans* have understandably focused on host traits, but there is also a significant body of work focusing on pathogen or parasite traits (*e.g.*, Tan *et al.* 1999; Sifri *et al.* 2003; Huber *et al.* 2004; White *et al.* 2016).

Theoretical characterizations of the genetics of host–pathogen relationships have focused on interactions between small numbers of host genes and pathogen genes (Frank 1994; Parker 1994; Agrawal and Lively 2002), so empirical investigations of the genetics of host susceptibility to infection have understandably focused on identifying genes of large effect. However, the prevalence of variation among host genotypes in pathogen susceptibility suggests that pathogen susceptibility may behave more like a classical polygenic trait than a Mendelian trait. Of note, Etienne *et al.* (2015) quantified the mutational input of genetic variation for susceptibility to the bacterial pathogen *Pseudomonas aeruginosa* in *C. elegans* and found that, indeed, mutational variance for susceptibility to *P. aeruginosa* accumulated at a rate similar to typical quantitative traits.

Genetic diversity was quantified for three toxin genes in the pathogen, and nine unlinked microsatellite loci in the host (Schulte *et al.* 2010). Results for the pathogen were clear: coevolution led to an increased rate of evolution, reduced diversity within populations, and greater diversity between populations (see also Masri *et al.* 2015). Results for the host were less clear-cut, with different loci exhibiting different patterns of evolution, although the overall rate of evolution was greater in the coevolved host than in the control. Coevolution led to an apparently increased rate of recombination in the pathogen but not in the host.

In one of the most influential coevolution experiments to date, morran and colleagues allowed experimental populations of *C. elegans* with varying degrees of outcrossing to coevolve with the bacterial pathogen *Serratia marcescens* (Morran *et al.* 2011). As briefly discussed above, the frequency of outcrossing increased in populations in which the pathogen was allowed to coevolve with the host, whereas populations of obligate selfers went extinct. Conversely, males were lost in populations in which the evolving *C. elegans* were exposed to non-coevolving pathogens. Similar results were observed in the pathogen, wherein coevolving populations evolved significantly greater infectivity than did populations evolving in the presence of a non-coevolving host. The results of these experiments are in concordance with predictions of the Red Queen hypothesis for the evolution of outcrossing (Jaenike 1978). However, the potential remains that Hill-Robertson effects resulting from fluctuating directional selection imposed by the pathogen rather than fluctuating epistatic selection resulting from negative frequency-dependent selection—the signature of the Red Queen (Barton 1995)—led to the advantage of outcrossing. More generally, it is unclear the extent to which sexual selection reverses, or reinforces, natural selection during coevolution, as different sexes can have different resistance and tolerance to pathogens (Masri *et al.* 2013).

How the composition of microbial communities can generate variable and fluctuating selection is, at present, unknown, and much work is needed in order to characterize these communities and their potential effects on *Caenorhabditis*. Are they constant during individual lifetime? Do they have high turnover rates when populations are challenged with novel environments? Two recent studies, by Dirksen *et al.* (2016) and Samuel *et al.* (2016), provided the first systematic sampling of the bacterial microbiota associated with field-collected *C. elegans*. Samuel and colleagues cultured *C. elegans* on >500 different bacterial isolates; there were consistent effects of bacterial taxon on nematode demography, and on multiple indicators of physiological stress. Dirksen *et al.* (2016) sampled the microbiomes associated with *C. elegans* and the congeneric *C. briggsae* and *C. remanei*, and reported consistent differences between the microbiotas of *C. remanei* and those of *C. elegans* and *C. briggsae*. Further, they found that populations of *C. elegans* initiated with a cocktail of 14 bacterial taxa consistently retained only a subset of taxa, but that the specific subset retained were specific to both (host) genotype and developmental stage.

## Future directions and conclusions

While *Caenorhabditis* EE has begun to touch on many aspects of population and quantitative genetics, its potential use in addressing questions related to evolution in structured populations remains largely untapped. An exception is the study of Gloria-Soria and Azevedo (2008) that showed that mutations at the *npr-1* locus could lead to differences in dispersal rates and the maintenance of polymorphism driven by behavioral characteristics. Another exception is the recent study of Greene *et al.* (2016), showing that mutations at the *srx-43* locus could lead to density-dependent selection. But few studies have used the systematic manipulation of migration rates to examine, say, the influence of local adaptation on rates of genomic change, or the evolution of dispersal traits (Friedenberg 2003). Indeed, a stack of Petri dishes, each containing its own population, would seem to be the perfect representation of S. wright’s original idealization of a collection of demes (Wright 1969).

Adding population structure to existing approaches would provide a nice complement of spatial variation in selection to previous studies that have largely concentrated on temporal variation in selection. As more environmental variables are added, investigation of transgenerational inheritance will also become increasingly important in *Caenorhabditis* EE studies, since experience of previous generations can clearly influence a wide variety of physiological and life history responses (Miska and Ferguson-Smith 2016). Caution is warranted to ensure that any work building upon our knowledge of transgenerational carry-over effects be conducted in a rigorous population genetic framework, as there is already a set of theory that deals with the genetics and selection for intergenerational interactions (*e.g.*, Lande and Price 1989; Lande and Kirkpatrick 1990; Slatkin 2009; Furrow and Feldman 2014; Proulx and Teotónio 2017). Part of the surprise from studies conducted to date, however, is that some of these effects appear to be extremely persistent, and, therefore, have the potential to obscure effects that would ordinarily be attributed to genetic changes.

In many cases, simply observing trait changes over time is sufficient for addressing the question at hand. Nevertheless, one of the strong appeals of using such well-developed model systems as *Caenorhabditis* for EE is the potential to identify the underlying genetic changes responsible for an evolutionary response—a goal quite apart from that of traditional mutagenesis studies. In this age of genomics, many candidate polymorphisms with potentially minor or rare contributions to a phenotype would be expected to emerge when millions of such polymorphisms are examined simultaneously. This can make it quite difficult to distinguish natural selection on individual loci from background levels of genetic drift across the whole genome. One of the primary advantages of *C. elegans* and its relatives over other metazoan models for EE is that it is reasonable to use very large population sizes, and, thus, to capture (and characterize) more such variants. Current studies frequently utilize thousands of individuals within a single population. Future experiments performed using liquid culture of *Caenorhabditis* or novel rearing approaches should allow populations to be maintained at sizes in the millions or more, making EE more typical of natural populations. While still perhaps not at yeast or bacteria population sizes, moving in this direction—especially in sexual species—has the potential to qualitatively change the way that experiments are conducted.

Once putative genetic changes have been identified, application of new genetic transformation methods will allow functional hypotheses to be more readily tested, especially outside of *C. elegans*. Perhaps more interestingly, the ability to make identical allelic substitutions in different genetic backgrounds opens up a new world of potential experiments. Chief among these will be the ability to more precisely test for epistatic effects between loci within different genetic backgrounds. One can readily imagine using CRISPR to manipulate the genetic backgrounds of different base populations that serve as the basis of later evolutionary experiments (Dickinson and Goldstein 2016). How dependent are evolutionary outcomes on initial genetic background? How are compensatory changes structured across different classes of mutations? What is the role of structural variation in determining the response to selection? These are a few of the kinds of questions that are now tractable with precision genomic editing.

Clearly, there are major gaps to be filled by future work aimed at identifying and characterizing the genetic and phenotypic bases of adaptation and by “translational” studies to connect such results to evolution in nature. Although functional genetic information for *Caenorhabditis* is still largely lacking (Petersen *et al.* 2015), some genetic factors mediating *C. elegans*’ tolerance to various forms of environmental challenges likely to be encountered by natural populations, such as pathogen exposure, or osmotic, thermal, and oxygen stress, have started to be dissected (*e.g.*, Lithgow *et al.* 1995; Lamitina *et al.* 2004; Frazier and Roth 2009; Reddy *et al.* 2009; LaRue and Padilla 2011; Andersen *et al.* 2014). Application of EE techniques wherein populations are exposed to such conditions would appear to hold great promise for uncovering the patterns, population genetic requirements, and genetic bases of adaptive responses (*e.g.*, Sikkink *et al.* 2014b, 2015; Dey *et al.* 2016).

Finally, the fundamental problem of evolution is the problem of the phenotype. One advantage of *Caenorhabditis* is that these nematodes are relatively easy to manipulate via a variety of methods, allowing high-throughput, high-precision phenotyping to be applied (Husson *et al.* 2013; Andersen *et al.* 2015). The potential of these approaches, *e.g.*, microfluidics (McCormick *et al.* 2011; Yan *et al.* 2014; Gupta and Rezai 2016), has not really begun to be utilized for EE. Similarly, the physical transparency of *Caenorhabditis* allows their cellular and developmental structure and function to be examined with exquisite detail in an EE context (Braendle *et al.* 2010; Farhadifar *et al.* 2015; Poullet *et al.* 2016). This opens the possibility creating a new and rigorous evolutionary cell and developmental biology, with *Caenorhabditis* as a central player (Braendle *et al.* 2011; Phillips and Bowerman 2015). The role that EE might play in such an effort is currently undefined.

*Caenorhabditis* species have come of age as models for EE. EE with these nematodes have provided significant insights into the origin and evolution of reproductive modes, adaptation to changing environments and into mutation rates, their genomic context and fitness effects. Use of *Caenorhabditis* allows unprecedented control over the properties of standing variation, population sizes, transgenerational effects, and degree of sexuality for a metazoan. Coupled with our extensive understanding of their genetics, and their cellular and developmental biology, future EE studies with *Caenorhabditis* promise to unravel many of the outstanding problems of evolutionary biology.

## Supplementary Material

Supplemental material is available online at www.genetics.org/lookup/suppl/doi:10.1534/genetics.115.186288/-/DC1.
