# Supplementary material for: Experimental Evolution with Caenorhabditis Nematodes
Source: Genetics. 2017;206(2):691–716. doi: 10.1534/genetics.115.186288 (PMC5499180; doi:10.1534/genetics.115.186288)
Supplement: Supplementary file 1 [file 691FileS1.pdf]

## Supplementary Materials for:

Experimental Evolution with *Caenorhabditis* Nematodes

Teotónio H., S. Estes, P.C. Phillips and C.F. Baer

**Appendix 1. Scaling and transformation in EE studies**. Comparisons of variances among treatments require that the traits be measured on comparable scales. How to achieve this is not a trivial problem (LYNCH and WALSH 1998). Mutational variances ($V_M$), and genetic variances in general ($V_G$), can be scaled in two ways, each of which having advantages and limitations. Traditionally, $V_M$ or $V_G$ is scaled relative to the within-line variance of the trait that has a stochastic environmental origin ($V_E$), due to measurement error and developmental instability. The ratio $V_M/V_E$ is called the "mutational heritability", and is analogous to the "narrow-sense heritability" of a trait, $V_A/V_E$ (where $V_A$ is the additive genetic variance, i.e. variation among individuals in their breeding value). The narrow-sense heritability predicts the extent to which the trait will respond to selection in the short-term from standing genetic variation, and similarly the mutational heritability predicts selection response from a situation without standing genetic variation. Alternatively, $V_M$ and $V_A$ can be scaled relative to the square of the trait mean, $V_M/\bar{z}^2$, which provides a sense for the long-term evolvability of the trait (HOULE 1992). In most circumstances scaling by the mean rather than by the variance is preferable (HANSEN *et al.* 2011), but in some circumstances scaling by the mean is not meaningful, as when the trait can take on values that are either positive or negative.

In MA experiments, it is customary to divide the trait variance of derived lines by the trait mean of the ancestral because this ancestral is fixed. Likewise, in EE from standing genetic variation, the trait mean of derived populations can be divided by the ancestral mean. But in cases where the trait mean evolves substantially, dividing by the

ancestral mean can give an unrealistically small estimate of the within population variance ($V_E$). In addition, since the ancestor mean trait value is taken as fixed, initial sampling of individuals to begin EE, particularly from standing genetic variation, must be large in order to avoid idiosyncratic responses. One solution is to divide each group (MA lines or derived populations and ancestor) by its own mean, which is equivalent to assuming that mutational or allelic trait effects are multiplicative. An alternative strategy is to log-transform the data before calculating the means and variances, which is almost equivalent to scaling by the treatment mean (FRY and HEINSOHN 2002). Yet an alternative is to repeat EE from several ancestors with different past histories, though how many are sufficient is an open question (WHITLOCK *et al.* 2002). Further note that having one or few ancestors necessarily makes experimental design unbalanced. Houle, Hansen, and their colleagues have written thoughtfully and in considerable depth about scaling in the context of evolutionary biology, and we strongly recommend that students of EE read their work on measurement theory (HOULE *et al.* 2011).

Yet another issue to consider in the design and interpretation of evolution experiments is the ability to detect low frequency genetic variants. The most straightforward and unbiased way to detect low frequency variants in multicellular organisms is to sequence the genomic DNA of multiple gametes taken from single individuals. Since the transcripts expressed in gametes are provided by the parent, the gamete's own genome is typically invisible to selection and thus any mutations present in the gamete genome are neutral to a first approximation and the mutational spectrum should be unbiased. However, there are practical difficulties involved in sequencing whole genomes of individual gametes. Isolating individual gametes is technically challenging and it is not yet possible to obtain whole-genome sequence from a single cell without an initial amplification step. The most accurate Taq polymerase has a per-

nucleotide error rate on the order of $10^{-6}$, whereas the per-nucleotide and per-generation mutation rate is on the order of $10^{-8}$ or less. PCR errors will thus outnumber real mutations by at least hundred-fold, but probably by much more. This problem can be avoided in principle by splitting the sample prior to the amplification step (if the amplification step does not itself introduce biases), but doing so obviously requires that each genome be sequenced twice. This can actually be accomplished fairly easily using an overlapping pair-end approach, which has been recently shown to be sensitive to very low frequency alleles in a *C. remanei* EE population (PRESTON *et al.* 2016).

Another method to detect low frequency variants is to sequence parents and offspring, or more generally, relatives within a pedigree that can be inferred. This is the technique by which human mutational properties have been characterized (CONRAD *et al.* 2011), and it can be done with individual *Caenorhabditis*, but an initial amplification step is still necessary to obtain enough material for whole-genome sequencing. A complicating factor is that, depending on when during the ontogeny of the parental germline a particular mutation occurred, the offspring may be either homozygous or heterozygous for a new mutation. The possibility that a new mutation segregates as a heterozygote adds the additional complication of binomial sampling. At any individual heterozygous nucleotide position, the probability of not sampling one of the two alleles is the binomial probability, $u = \binom{N}{0} p(1-p)^N$, where $N$ is the number of times the nucleotide position is represented in the data (the coverage) and $p$ is 0.5 (the frequency of the allele). To achieve a 95% probability that *no* heterozygous site is misidentified as homozygous – or in other words, $(1\text{-}u)^X > 0.95$, where $X$ is $10^8$ - a genome of $10^8$ bases needs to be sequenced to at least 31X *minimum* coverage, or at least 36X to identify each allele at least twice, and thereby be able to distinguish a true mutation from a sequencing error.

One last consideration that is relevant in any kind of evolution experiment is how many genomes are actually being sequenced? As noted, sequencing single individuals, and thus single genomes, requires an amplification step. To date, sequencing *Caenorhabditis* genomes involves growing up a large population of individuals, extracting their DNA and sequencing the population sample. How much of a problem does sequencing from a population rather than individuals cause? Consider a *C. elegans* population taken from a single 100 cm plate after two generations of population expansion. If the population is initiated from a single hermaphrodite who produces 200 offspring, each of whom has 200 offspring, that means there are 40,000 individuals on the plate from which DNA is extracted (40,000 is probably an overestimate). If the haploid genomic mutation rate is one per generation, the initial individual is expected to be heterozygous for new mutations at two loci; the expected frequency of those alleles in the sequencing population is 0.5. Each of the 200 offspring is expected to be heterozygous at two loci, which means we expect 400 new mutant alleles, each at an expected frequency of 1/400. Obviously, only the couple of mutations that arise in the initial individual could potentially be characterized as mutations.

**Appendix 2. Two examples of competition experiments.** Short-term competition experiments can be employed to test the environmental conditions under which a given mutant of interest or natural variant might be selected. In *C. elegans*, for instance, the now classic competition experiments of Walker and colleagues (WALKER *et al.* 2000) showed that the *age-1(hx546)* mutant allele, originally described as increasing hermaphrodite lifespan of up to 60% relative to the wildtype allele (FRIEDMAN and JOHNSON 1988), was quickly selected against when in competition over six starvation cycles (about 20 generations). Conversely, when competed in non-limiting food conditions, both mutant and wildtype alleles were stably maintained. The *age-1(hx546)* allele thus seems to impose a fitness cost under starvation conditions, suggesting that evolution of aging can occur by selection on pleiotropic alleles with antagonistic effects between early- and late-life traits, such as early fecundity and lifespan. In agreement with the evolutionary theory of aging (WILLIAMS 1957; HAMILTON 1966), the existence of a trade-off between fitness components under certain environments can underlie the evolution of aging in *C. elegans*.

In a more recent example, competition experiments were used to show that a deletion allele in the RNAi pathway *drh-1* locus is selectively disfavored when *C. elegans* hermaphrodites are infected with the RNA Orsay virus, while showing no cost when animals are uninfected (ASHE *et al.* 2013). Interestingly, the deletion allele did not go extinct over the course of 16 generations and was stably maintained at about 10% after 8 generations. Selection against the *drh-1* deletion allele during the first half of the experiment is partly explained by delayed and decreased fecundity when hermaphrodites are infected, so it is puzzling why it appeared to be neutral during the second half of the experiment and why it is found at intermediate frequencies of 20% to 30% among wild isolates (ASHE *et al.* 2013). Since wild isolates show high linkage

disequilibrium in the center region of chromosome IV, where the *drh-1* locus is located and where recombination rates are low (ROCKMAN and KRUGLYAK 2009), it could be that the deleterious deletion allele partially spread by hitch-hiking with an uncharacterized beneficial allele at another location (ROCKMAN *et al.* 2010; ANDERSEN *et al.* 2012).

Another explanation for why alternative *drh-1* alleles were maintained at intermediate frequencies during the second half of the experiment is frequency- and/or density-dependent selection. In *C. elegans*, for example, (CHELO *et al.* 2013) showed that an inbred line that outcompeted another inbred line at low and high frequencies was strongly disfavored at intermediate frequencies. The fact that both inbred lineages were derived from a laboratory adapted population could in part explain the frequency-dependence and the balancing selection among the inbred lineages. Such a scenario is predicted from evolutionary theory where, with laboratory adaptation, overdominance for fitness is expected to become common since stabilizing selection for intermediate phenotypic trait optima becomes more predominant relative to directional selection.

**Appendix 3. Design and interpretation of Mutation Accumulation (MA) experiments, and some findings about the mutational process in *Caenorhabditis*.**

The principle of an MA experiment is simple: minimize the efficiency of selection, so that all but the most severely deleterious mutations are invisible to selection. An MA experiment is a pedigree in which the lineages ("MA lines") are the (great)$^t$-grandchildren of an isogenic[1] founding ancestor, with $t$ being the number of generations since the ancestor. The initially genetically-identical MA line genomes will diverge as each one accumulate its own unique set of mutations. Selection is minimized by minimizing $N_e$; mutations with selective effects $s < 1/4N_e$ are approximately neutral (KIMURA 1962; KEIGHTLEY and CABALLERO 1997) and will accumulate at approximately the neutral rate. In a population of self-fertile hermaphrodites, such as *C. elegans*, the minimum $N_e = 1$.

At the phenotypic level, MA experiments provide two measurable outputs. First, comparison of the mean phenotype of the MA lineages ($\bar{Z}_{MA}$) with the mean phenotype of the unevolved ancestor ($\bar{Z}_0$) provides an estimate of the rate at which the trait changes under mutational pressure. Scaled per-generation as a fraction of the ancestral trait mean, this quantity is sometimes referred to as the "mutational bias" $\Delta M = \frac{(\bar{Z}_{\mathrm{MA}} - \bar{Z}_0)}{t\bar{Z}_0}$, where $t$ is the number of generations of MA. $\Delta M$ can be decomposed into its components, $\Delta M = \mu_G \alpha$, where $\mu_G$ is the mutation rate per-genome per-generation, and $\alpha$ is the average effect of a mutation on the trait.

---

[1] There is another method of mutation accumulation, the so-called "Middle Class Neighborhood" method SHABALINA, S. A., L. YAMPOLSKY and A. S. KONDRASHOV, 1997 Rapid decline of fitness in panmictic populations of Drosophila melanogaster maintained under relaxed natural selection. PNAS **94:** 13034-13039.which mutations are allowed to accumulate from a genetically variable progenitor population. In the MCN method, selection is minimized by eliminating the variance in family size. The MCN design has not been used with *Caenorhabditis*.

The second measurable output from MA data is the increase in the among-line variance, $\Delta V_L$. Since the ancestor is isogenic, initially there will be (almost) no segregating genetic variation. Once the population is divided into MA lines, mutations accumulate independently in each MA line at rate $\mu_G$ per genome per generation, and each mutation contributes to the genetic variance with expectation $\alpha^2$. The per-generation increase of genetic variance due to new mutations is $V_M = \mu_G\alpha^2$. MA lines diverge at rate $2V_M$ per generation, which is equivalent to saying that $V_L$ increases at $2V_M$/generation, so $V_L/2t$ provides an estimate of $V_M$ (LYNCH and WALSH 1998, p. 330).

MA experiments have been employed in *Caenorhabditis* to estimate the cumulative effects of mutations at the phenotypic level and to reveal their molecular spectrum. Fitness assays can be used to estimate the genome-wide rate and average fitness effect of accumulated mutations, an exercise that confirms the deleterious nature of most new mutations (KEIGHTLEY and CABALLERO 1997; VASSILIEVA and LYNCH 1999), and suggests that, on average, their individual consequences for fitness may be relatively minor, with the majority of mutational variance for fitness contributed by a few mutations with large effects (TURELLI 1984; KONDRASHOV and KONDRASHOV 2010). However, because the fitness effects of individual mutations are not characterized directly in standard MA experiments, the true shape of the distribution of effects cannot be revealed by this approach. Similarly, information on mutational properties including dominance effects, the nature of epistatic interactions, the effect of genetic background and environment, in addition to potential species differences in mutation rate is also largely lacking (BAER *et al.* 2005; SALOMON *et al.* 2009; ESTES *et al.* 2011; DENVER *et al.* 2012; PHILLIPS *et al.* 2015).

While knowledge of the cumulative mutational variation coming into a population each generation is useful, what we would really like to know is the full

distribution of mutational effects (DME), particularly the distribution of fitness effects (DFE) (EYRE-WALKER and KEIGHTLEY 2007). Since $4N_e s < 1$ approximately demarcates the threshold of effective neutrality, one way to characterize the DFE is by manipulating $N_e$. The expectation is that the cumulative effects of retained deleterious mutations should decrease at a rate proportional to the population size. S. ESTES, V. KATJU, and their colleagues applied this approach to approximate the DFE of new mutations in *C. elegans* (ESTES *et al.* 2004; KATJU *et al.* 2015). In both experiments, almost all of the decline in fitness could be attributed to mutations with large effects. Specifically, lineages maintained in bottleneck sizes of only 5 or 10 were able to maintain high levels of fitness across generations in both experiments, while lineages maintained at $N_e$=1 or 2 exhibited the expected decline in mean fitness and increase in among-line variance. However, ESTES ET AL. (2004) did find evidence of classes of mutations with very small deleterious effects or with no measureable effects at all implying that the true distribution of mutational effects for well-adapted populations may be bimodal, with some new mutations having strongly detrimental effects and more having nearly-neutral or neutral individual effects. Mildly deleterious mutations can readily accumulate within populations, ultimately leading to extinction ("mutational meltdown"; (LYNCH 1993; LYNCH *et al.* 1995).

In the era of whole-genome sequencing, MA experiments provide a cost-effective way to infer mutational properties at the level of the genome itself. (DENVER *et al.* 2000) reported the first direct estimate of the mutational properties of the mitochondrial genome in any organism, based on sequencing the mtDNA of a set of N2 strain MA lines. In 2004, the same group reported the first direct estimate of the nuclear genome-wide mutation rate in any multicellular organism (DENVER *et al.* 2004b). Additional studies have built on that work, including characterizations of the mtDNA

and nuclear base-substitution spectrum in other strains and species (HOWE *et al.* 2010; DENVER *et al.* 2012) and features of the genome beyond base-substitutions, including short tandem repeats (DENVER *et al.* 2004a; SEYFERT *et al.* 2008; PHILLIPS *et al.* 2009) and large structural variants (LIPINSKI *et al.* 2011). (GOUT *et al.* 2013) devised a novel method to detect mutations in RNA transcripts and applied the method in *C. elegans*, providing the first direct estimate of the transcriptional mutation rate at genome-wide scale.

**Appendix 4. Population genetic consequences of selfing.** Variation in selfing rate, be it intended or accidental, may have significant implications for the design and interpretation of EE. The main effect of selfing on population genetics is to change the expected distributions of gametic frequencies relative to random mating by increasing the probability of identity-by-descent between any two alleles in embryos. For single neutral loci, heterozygosity at time *t* under selfing is $H_t = 0.5H_{t-1}$ (CROW and KIMURA 1970). A fixation index (*F*) can be estimated as the deviation of observed ($H_o$) from expected heterozygosity under random mating ($H_e$) as $F=1- (H_o/H_e)$ and interpreted as an inbreeding coefficient. While heterozygosity will be reduced relative to the ancestral population, differentiation among populations under selfing will nonetheless be larger than under random mating, after a sufficient time being $1+F$ times the heterozygosity of the ancestral. In partially selfing populations at equilibrium, the fixation index *F* is related to the selfing rate *S* of a population by $F= S/(2-S)$. Since *Caenorhabditis* hermaphrodites cannot outcross with each other, $S=1-2m$, where *m* is the male proportion, is a good approximation of selfing rate (STEWART and PHILLIPS 2002; CUTTER 2004). Mutation to males can usually be ignored. *Caenorhabditis* are diploid, with hermaphrodites or females XX and males X0. Males can be produced by mutation through the non-disjunction of the X-chromosome during hermaphrodite gametogenesis (NIGON 1949; HODGKIN *et al.* 1979), but their appearance in natural isolates is relatively rare, on the order of $10^{-3}$-$10^{-4}$ (TEOTÓNIO *et al.* 2006; TEOTÓNIO *et al.* 2012).

Population genetic dynamics of multiple loci under (partial) selfing are highly complex and still poorly understood when they depend on the degree of dominance, epistasis, and linked selection (e.g., (WEIR *et al.* 1980; ZIEHE and ROBERDS 1989; ROZE 2015; ROZE 2016). For neutral loci unlinked to any selected alleles, the effective population size under partial selfing and at genetic drift-mutation equilibrium is

expected to be $N_e = N/(1+F)=N(2-S)/2$ (POLLAK 1987; NORDBORG 2000), where $N$ is population size. Selfing is expected to halve heterozygosity according to $H_t = H_{t0} (1-S) + S/2 (H_t-1)$, with $H_{t0}$ being the observed heterozygosity of the population at time $t=0$ before selfing started. Heterozygosity will only be a fraction $1-F = 1-1/2N_e =1-1/N(2-S)$ of the ancestral levels, with great loss of heterozygosity and concomitant increase in homozygosity occurring only if relatively high selfing rates are maintained for long periods. At the genome-wide level, selfing increases "identity disequilibrium" between loci, by increasing homozygosity correlations among any two neutral loci as: $G=[4S(1-S)\{1+\lambda^2(1-S)\}]/[(2-S)^2\{4-S(1+\lambda^2)\}]$, where $\lambda=1-2r$ and $r$ recombination rate (WEIR and COCKERHAM 1973; CHRISTIANSEN 1989).

As a consequence of increased homozygosity at multiple loci, selfing is also expected to decrease the "effective" recombination rates ($r_s$) by the fraction $1-1/N(2-S)$ (NORDBORG 1997; NORDBORG 2000). An important application of this model is to measure historical selfing rates (NORDBORG and DONNELLY 1997), which has indicated that natural populations of *C. elegans* have predominantly inbred by selfing for thousands of generations (BARRIERE and FELIX 2005). In particular, assuming an equilibrium between recombination and genetic drift, the expected average correlation of allele diversity between two neutral loci—$r^2$, a linkage disequilibrium metric that can be estimated by sampling genotypes—is $E(r^2)=1/1+4N_er_s$ (HUDSON and KAPLAN 1988) (HUDSON and KAPLAN 1988). This relationship shows that effective recombination will be little affected unless selfing is predominant for a long time or effective population sizes are relatively small. Consistent with this theoretical prediction, (CHELO and TEOTÓNIO 2013) compared the genotype dynamics of obligate outcrossing dioecious *C. elegans* populations with those of partially-selfing androdioecious populations during 100 generations of EE, and found that $r^2$ for both

population types decayed with physical distance between SNPs mostly as expected under random mating. (CHELO and TEOTÓNIO 2013) did, however, find that the partially-selfing populations generated ~0.5% fewer new haplotypes per generation as compared to obligate outcrossing populations. Furthermore, the appearance of new genotypes was non-random across time and genomic location, suggesting that the interaction between selection and effective recombination cannot be ignored. The distribution of $r^2$ across the genome is surely highly variable, especially for short physical distances, and its accurate description requires very high densities of SNPs or other DNA-based polymorphic markers.

Similar to its effect on segregation and recombination, selfing is expected to reduce the overall size of the **G**-matrix -- the matrix whose entries are the additive genetic variances and covariances between traits (LANDE 1980; PHILLIPS and MCGUIGAN 2006) -- by a fraction $1\text{-}F=1\text{-}1/N(2\text{-}S)$ of the ancestral size while causing no change in **G**-matrix orientation on average. After a period of selfing sufficient to achieve complete homozygosity, additive genetic variances will be composed of all but strictly genetic variance that can be estimated from trait differentiation among selfing lineages (LYNCH and WALSH 1998). However, the expected genetic variance between selfing populations differentiating only by genetic drift will be $2F=2/N(2\text{-}S)$ times the genetic variation of the ancestral population (assumed to be panmictic and outcrossing); again reflecting the higher segregation of homozygotes when compared to randomly mating populations. This has the important consequence for EE design since more replication is necessary under selfing than outcrossing to detect an evolutionary trait response of similar magnitude.

**Table S1. EE resources in *C. elegans*.**

| Purpose | Material | Reference |
|---|---|---|
| Ancestral populations | Hybrids between wild isolates | (ANDERSON *et al.* 2010; SCHULTE *et al.* 2010; TEOTÓNIO *et al.* 2012) |
| | Mutagenized wild isolates with variable sex ratios | (CUTTER 2005; MANOEL *et al.* 2007; MORRAN *et al.* 2009) |
| | ~60 250-generation MA lines ($N_e$≈1) from N2 and PB306 (*C. elegans*) and PB800 and HK104 (*C. briggsae*) [Baer lab]. Also ~35 400-generation N2 MA lines (20 $N_e$≈1, 10 $N_e$≈10, 5 $N_e$≈100) [Katju lab]. | (BAER *et al.* 2005; KATJU *et al.* 2015) |
| Domesticated/lab adapted populations | N2, LSJ2 strains | (MCGRATH *et al.* 2011) |
| | Populations with standing genetic variation and variable sex ratios | (THEOLOGIDIS *et al.* 2014) |
| Testers | GFP under a *myo-3* promoter introgressed into ancestral and lab adapted populations | (TEOTÓNIO *et al.* 2012; CHELO *et al.* 2013; THEOLOGIDIS *et al.* 2014) |
| | Mutant strains of sex determination, such as *fog-2(q71)* in the N2 background or in lab adapted population | *Caenorhabditis* Genetics Center, (THEOLOGIDIS *et al.* 2014) |
| QTL panels | Advanced F2 recombinant inbred lines (RILs) between N2 and non-domesticated strains or other biparental crosses | (ROCKMAN and KRUGLYAK 2009; DUVEAU and FELIX 2012; ANDERSEN *et al.* 2015; NOBLE *et al.* 2015) |
| | Nearly isogenic lines (NILs) from N2 and CB4856 | (DOROSZUK *et al.* 2009) |
| | ~250 “isotypes” from more than 900 wild isolates sequenced | (COOK *et al.* 2017) |
| | ~500 RILs from lab adapted populations sequenced | (NOBLE *et al.* 2017) |
| Protocols | Lab culturing | (STIERNAGLE 1999) |

| | | |
|---|---|---|
| | Scripts for population genetics simulations | (DENVER *et al.* 2010; CHELO and TEOTÓNIO 2013; NOBLE *et al.* 2017), archived in *Dryad.org* |
| Community | Dedicated websites and databases | http://evolution.wormbase.org, http://www.justbio.com/worms/index.php, https://elegansvariation.org/ |

## Literature Cited


ANDERSEN, E. C., J. P. GERKE, J. A. SHAPIRO, J. R. CRISSMAN, R. GHOSH *et al.*, 2012 Chromosome-scale selective sweeps shape Caenorhabditis elegans genomic diversity. Nat Genet **44:** 285-290.

ANDERSEN, E. C., T. C. SHIMKO, J. R. CRISSMAN, R. GHOSH, J. S. BLOOM *et al.*, 2015 A Powerful New Quantitative Genetics Platform, Combining Caenorhabditis elegans High-Throughput Fitness Assays with a Large Collection of Recombinant Strains. G3 (Bethesda) **5:** 911-920.

ANDERSON, J. L., L. T. MORRAN and P. C. PHILLIPS, 2010 Outcrossing and the maintenance of males within C. elegans populations. J Hered **101 Suppl 1:** S62-74.

ASHE, A., T. BÉLICARD, J. LE PEN, P. SARKIES, L. FRÉZA *et al.*, 2013 A deletion polymorphism in the Caenorhabditis elegans RIG-I homolog disables viral RNA dicing and antiviral immunity. eLife.

BAER, C. F., F. SHAW, C. STEDING, M. BAUMGARTNER, A. HAWKINS *et al.*, 2005 Comparative evolutionary genetics of spontaneous mutations affecting fitness in rhabditid nematodes. Proc Natl Acad Sci U S A **102:** 5785-5790.

BARRIERE, A., and M. A. FELIX, 2005 High local genetic diversity and low outcrossing rate in Caenorhabditis elegans natural populations. Curr Biol **15:** 1176-1184.

CHELO, I. M., J. NÉDLI, I. GORDO and H. TEOTÓNIO, 2013 An experimental test on the probability of extinction of new genetic variants. Nature Communications **4:** 10.1038/ncomms3417.

CHELO, I. M., and H. TEOTÓNIO, 2013 The opportunity for balancing selection in experimental populations of Caenorhabditis elegans. Evolution **67:** 142-156.

CHRISTIANSEN, F. B., 1989 Linkage equilibrium in multi-locus genotypic frequencies with mixed selfing and random mating. Theor Pop Biol **35:** 307-336.

CONRAD, D. F., J. E. M. KEEBLER, M. A. DEPRISTO, S. J. LINDSAY, Y. ZHANG *et al.*, 2011 Variation in genome-wide mutation rates within and between human families. Nat Genet **43:** 712-714.

COOK, D. E., S. ZDRALJEVIC, J. P. ROBERTS and E. C. ANDERSEN, 2017 CeNDR, the Caenorhabditis elegans natural diversity resource. Nucleic Acids Res **45:** D650-D657.

CROW, J. F., and M. KIMURA, 1970 *An Introduction to Population Genetics Theory*. Harper & Row, Publishers, New York.

CUTTER, A. D., 2004 Sperm-limited fecundity in nematodes: how many sperm are enough? Evolution **58:** 651-655.

CUTTER, A. D., 2005 Mutation and the experimental evolution of outcrossing in Caenorhabditis elegans. J Evol Biol **18:** 27-34.

DENVER, D. R., D. K. HOWE, L. J. WILHELM, C. A. PALMER, J. L. ANDERSON *et al.*, 2010 Selective sweeps and parallel mutation in the adaptive recovery from deleterious mutation in Caenorhabditis elegans. Genome Res **20:** 1663-1671.

DENVER, D. R., K. MORRIS, A. KEWALRAMANI, K. E. HARRIS, A. CHOW *et al.*, 2004a Abundance, distribution, and mutation rates of homopolymeric nucleotide runs in the genome of Caenorhabditis elegans. J Mol Evol **58:** 584-595.

DENVER, D. R., K. MORRIS, M. LYNCH and W. KELLEY THOMAS, 2004b High mutation rate and predominance of insertions in the Caenorhabditis elegans nuclear genome. Nature **430:** 679-682.

DENVER, D. R., K. MORRIS, M. LYNCH, L. L. VASSILIEVA and W. K. THOMAS, 2000 High Direct Estimate of the Mutation Rate in the Mitochondrial Genome of Caenorhabditis elegans. Science **289:** 2342-2344.

DENVER, D. R., L. J. WILHELM, D. K. HOWE, K. GAFNER, P. C. DOLAN *et al.*, 2012 Variation in base-substitution mutation in experimental and natural lineages of Caenorhabditis nematodes. Genome Biol Evol **4:** 513-522.

DOROSZUK, A., L. B. SNOEK, E. FRADIN, J. RIKSEN and J. KAMMENGA, 2009 A genome-wide library of CB4856/N2 introgression lines of Caenorhabditis elegans. Nucleic Acids Res **37:** e110.

DUVEAU, F., and M. A. FELIX, 2012 Role of pleiotropy in the evolution of a cryptic developmental variation in Caenorhabditis elegans. PLoS Biol **10:** e1001230.

ESTES, S., P. C. PHILLIPS and D. R. DENVER, 2011 Fitness recovery and compensatory evolution in natural mutant lines of C. elegans. Evolution **65:** 2335-2344.

ESTES, S., P. C. PHILLIPS, D. R. DENVER, W. K. THOMAS and M. LYNCH, 2004 Mutation accumulation in populations of varying size: the distribution of mutational effects for fitness correlates in Caenorhabditis elegans. Genetics **166:** 1269-1279.

EYRE-WALKER, A., and P. D. KEIGHTLEY, 2007 The distribution of fitness effects of new mutations. Nat Rev Genet **8:** 610-618.

FRIEDMAN, D. B., and T. E. JOHNSON, 1988 A Mutation in the age-1 Gene in Caenorhabditis elegans lengthens Life and Reduces Hermaphrodite Fertility. Genetics **118:** 75-86.

FRY, J. D., and S. L. HEINSOHN, 2002 Environment Dependence of Mutational Parameters for Viability in Drosophila melanogaster. Genetics **161:** 1155–1167.

GOUT, J. F., W. K. THOMAS, Z. SMITH, K. OKAMOTO and M. LYNCH, 2013 Large-scale detection of in vivo transcription errors. Proceedings of the National Academy of Sciences **110:** 18584-18589.

HAMILTON, W. D., 1966 The moulding of senescence by natural selection. J. Theor. Biol. **12:** 12-45.

HANSEN, T. F., C. PÉLABON and D. HOULE, 2011 Heritability is not Evolvability. Evolutionary Biology **38:** 258-277.

HODGKIN, J., H. R. HORVITZ and S. BRENNER, 1979 Nondisjunction mutants of the nematode Caenorhabditis elegans. Genetics **91:** 67-94.

HOULE, D., 1992 Comparing evolvability and variability of quantitative traits. Genetics **130:** 195-204.

HOULE, D., C. PÉLABON, G. P. WAGNER and T. F. HANSEN, 2011 Measurement and meaning in biology. Quaterly Review of Biology **86:** 3-34.

HOWE, D. K., C. F. BAER and D. R. DENVER, 2010 High rate of large deletions in Caenorhabditis briggsae mitochondrial genome mutation processes. Genome Biol Evol **2:** 29-38.

HUDSON, R. R., and N. L. KAPLAN, 1988 The coalescent process in models with selection and recombination. Genetics **120:** 831-840.

KATJU, V., L. B. PACKARD, L. BU, P. D. KEIGHTLEY and U. BERGTHORSSON, 2015 Fitness decline in spontaneous mutation accumulation lines of Caenorhabditis elegans with varying effective population sizes. Evolution **69:** 104-116.

KEIGHTLEY, P. D., and A. CABALLERO, 1997 Genomic mutation rates for lifetime reproductive output and lifespan in Caenorhabditis elegans. PNAS **94:** 3823–3827.

KIMURA, M., 1962 On the probability of fixation of mutant genes in a population. Genetics **47:** 713–719.

KONDRASHOV, F. A., and A. S. KONDRASHOV, 2010 Measurements of spontaneous rates of mutations in the recent past and the near future. Philosophical Transactions of the Royal Society B: Biological Sciences **365:** 1169-1176.

LANDE, R., 1980 The Genetic Covariance between Characters Maintained by Pleiotropic Mutations. Genetics **94:** 203-215.

LIPINSKI, K. J., J. C. FARSLOW, K. A. FITZPATRICK, M. LYNCH, V. KATJU *et al.*, 2011 High Spontaneous Rate of Gene Duplication in Caenorhabditis elegans. Current Biology **21:** 306-310.

LYNCH, M., 1993 The mutational meltdown in asexual populations. . J Hered **84:** 339-340.

LYNCH, M., J. CONERY and R. BURGER, 1995 Mutation accumulation and extinction in small populations. Am Nat **146:** 489-518.

LYNCH, M., and B. WALSH, 1998 *Genetics and Analysis of Quantitative Traits*. Sinauer Associates, Inc., Sunderland.

MANOEL, D., S. CARVALHO, P. C. PHILLIPS and H. TEOTÓNIO, 2007 Selection against males in Caenorhabditis elegans under two mutational treatments. Proc Biol Sci **274:** 417-424.

MCGRATH, P. T., Y. XU, M. AILION, J. L. GARRISON, R. A. BUTCHER *et al.*, 2011 Parallel evolution of domesticated Caenorhabditis species targets pheromone receptor genes. Nature **477:** 321-325.

MORRAN, L. T., M. D. PARMENTER and P. C. PHILLIPS, 2009 Mutation load and rapid adaptation favour outcrossing over self-fertilization. Nature **462:** 350-352.

NIGON, V., 1949 Les modalités de la reproduction et le déterminisme de sexe chez quelques nematodes libres. Ann. Sci. Natur. Zool. **2:** 1-132.

NOBLE, L., I. M. CHELO, T. GUZELLA, B. AFONSO, D. RICCARDI *et al.*, 2017 Polygenicity and epistasis underlie fitness-proximal traits in the Caenorhabditis elegans multiparental experimental evolution (CeMEE) panel. biorxiv.org**:** doi:10.1101/120865.

NOBLE, L. M., A. S. CHANG, D. MCNELIS, M. KRAMER, M. YEN *et al.*, 2015 Natural Variation in plep-1 Causes Male-Male Copulatory Behavior in C. elegans. Curr Biol.

NORDBORG, M., 1997 Structured coalescent processes on different time scales. Genetics **146:** 1501-1514.

NORDBORG, M., 2000 Linkage disequilibrium, gene trees and selfing: an ancestral recombination graph with partial self-fertilization. Genetics **154:** 923-929.

NORDBORG, M., and P. DONNELLY, 1997 The coalescent process with selfing. Genetics.

PHILLIPS, N., M. SALOMON, A. CUSTER, D. OSTROW and C. F. BAER, 2009 Spontaneous mutational and standing genetic (co)variation at dinucleotide microsatellites in Caenorhabditis briggsae and Caenorhabditis elegans. Mol Biol Evol **26:** 659-669.

PHILLIPS, P., and K. MCGUIGAN, 2006 Evolution of Genetic Variance-Covariance Structure in *Evolutionary Genetics: Concepts and Case Studies*, edited by C. W. F. A. J. B. WOLF. Oxford University Press, Oxford.

PHILLIPS, W. S., A. L. COLEMAN-HULBERT, E. S. WEISS, D. K. HOWE, S. PING *et al.*, 2015 Selfish Mitochondrial DNA Proliferates and Diversifies in Small, but not Large, Experimental Populations of Caenorhabditis briggsae. Genome Biol Evol **7:** 2023-2037.

POLLAK, E., 1987 On the theory of partially inbreeding finite populations. I. Partial selfing. Genetics **117:** 353-360.

PRESTON, J. L., A. E. ROYALL, M. A. RANDEL, K. L. SIKKINK, P. C. PHILLIPS *et al.*, 2016 High-specificity detection of rare alleles with Paired-End Low Error Sequencing (PELE-Seq). BMC Genomics **16**.

ROCKMAN, M. V., and L. KRUGLYAK, 2009 Recombinational landscape and population genomics of Caenorhabditis elegans. PLoS Genet **5:** e1000419.

ROCKMAN, M. V., S. S. SKROVANEK and L. KRUGLYAK, 2010 Selection at linked sites shapes heritable phenotypic variation in C. elegans. Science **330:** 372-376.

ROZE, D., 2015 Effects of Interference Between Selected Loci on the Mutation Load, Inbreeding Depression, and Heterosis. Genetics **201:** 745–757.

ROZE, D., 2016 Background selection in partially selfing populations. Genetics **203:** 937-957.

SALOMON, M. P., D. OSTROW, N. PHILLIPS, D. BLANTON, W. BOUR *et al.*, 2009 Comparing mutational and standing genetic variability for fitness and size in Caenorhabditis briggsae and C. elegans. Genetics **183:** 685-692, 681SI-619SI.

SCHULTE, R. D., C. MAKUS, B. HASERT, N. K. MICHIELS and H. SCHULENBURG, 2010 Multiple reciprocal adaptations and rapid genetic change upon experimental coevolution of an animal host and its microbial parasite. Proc Natl Acad Sci U S A **107:** 7359-7364.

SEYFERT, A. L., M. E. CRISTESCU, L. FRISSE, S. SCHAACK, W. K. THOMAS *et al.*, 2008 The rate and spectrum of microsatellite mutation in Caenorhabditis elegans and Daphnia pulex. Genetics **178:** 2113-2121.

SHABALINA, S. A., L. YAMPOLSKY and A. S. KONDRASHOV, 1997 Rapid decline of fitness in panmictic populations of Drosophila melanogaster maintained under relaxed natural selection. PNAS **94:** 13034-13039.

STEWART, A. D., and P. C. PHILLIPS, 2002 Selection and maintenance of androdioecy in Caenorhabditis elegans. Genetics **160:** 975-982.

STIERNAGLE, T., 1999 *Maintenance of C. elegans*. Oxford University Press, Oxford.

TEOTÓNIO, H., S. CARVALHO, D. MANOEL, M. ROQUE and I. M. CHELO, 2012 Evolution of outcrossing in experimental populations of Caenorhabditis elegans. PLoS One **7:** e35811.

TEOTÓNIO, H., D. MANOEL and P. C. PHILLIPS, 2006 Genetic variation for outcrossing among Caenorhabditis elegans isolates. Evolution **60:** 1300-1305.

THEOLOGIDIS, I., I. M. CHELO, C. GOY and H. TEOTÓNIO, 2014 Reproductive assurance drives transitions to self-fertilization in experimental Caenorhabditis elegans. BMC Biology **12:** 93.

TURELLI, M., 1984 Heritable genetic variation via mutation-selection balance: Lerch's zeta meets the abdominal bristle. Theor Pop Biol **25:** 138-193.

VASSILIEVA, L. L., and M. LYNCH, 1999 The rate of spontaneous mutation for life-history traits in Caenorhabditis elegans. Genetics **151:** 119-129.

WALKER, D. W., MCCOLL, N. L. JENKINS, J. HARRIS and G. J. LITHGOW, 2000 Evolution of lifespan in C. elegans. Nature **405:** 296-297.

WEIR, B., and C. C. COCKERHAM, 1973 Mixed selfing and random mating at two loci. Genetical Research **21:** 247-262.

WEIR, B. S., P. J. AVERY and W. G. HILL, 1980 Effect of mating structure on variation in inbreeding. Theor Pop Biol **18:** 396-429.

WHITLOCK, M. C., P. C. PHILLIPS and K. FOWLER, 2002 Persistence of changes in the genetic covariance matrix after a bottleneck. Evolution **56:** 1968-1975.

WILLIAMS, G. C., 1957 Pleiotropy, Natural Selection, and the Evolution of Senescence. Evolution **11:** 398-411.

ZIEHE, M., and J. H. ROBERDS, 1989 Inbreeding depression due to overdominance in partially self-fertilizing plant populations. Genetics **121:** 861-868.
